# Signs, symptoms, and health-related quality of life in MELAS: measuring what’s important from the patient and clinician perspectives

**DOI:** 10.1186/s41687-025-00962-6

**Published:** 2025-10-27

**Authors:** Paolo Medrano, Benjamin Banderas, Marisa Brimmer, Lily Settel, Sari Berger, Alan Shields, Amy Goldstein, Amel Karaa, Austin Larson, Sumit Parikh, Fernando Scaglia, Karra Danyelle Harrington, Chris James Edgar, Pamela Ventola, Matthew Webster, Jennifer Chickering, Chad Gwaltney, Phebe Wilson, Chad Glasser

**Affiliations:** 1 Adelphi Values, One Lincoln Street, Suite 2900, Boston, MA 02111 USA; 2 Mitochondrial Medicine Frontier Program, Division of Human Genetics, Department of Pediatrics, The Children’s Hospital of Philadelphia, Philadelphia, PA 19104 USA; 3 Department of Pediatrics, University of Pennsylvania Perelman School of Medicine, Philadelphia, PA 19104 USA; 4 Division of Genetics Unit, Department of Pediatrics, Massachusetts General Hospital, Harvard Medical School, Boston, MA USA; 5 Section of Clinical Genetics and Metabolism, Department of Pediatrics, University of Colorado, Aurora, CO 80045 USA; 6 Mitochondrial Medicine Center, Neurosciences Institute, Cleveland, OH USA; 7 Department of Molecular and Human Genetics, Baylor College of Medicine, Houston, TX USA; 8 Texas Children’s Hospital, Houston, TX USA; 9 Joint BCM-CUHK Center of Medical Genetics, Prince of Wales Hospital, Shatin, Hong Kong SAR China; 10 Cogstate Ltd., New Haven, CT USA; 11 Tisento Therapeutics, Cambridge, MA USA; 12 Gwaltney Consulting, Westerly, RI USA

**Keywords:** Mitochondrial encephalomyopathy, lactic acidosis, and stroke-like episodes, MELAS, Mitochondrial disease, Concept elicitation, Qualitative interviews, Patient-centered outcomes

## Abstract

**Background and objectives:**

Mitochondrial encephalomyopathy with lactic acidosis and stroke-like episodes (MELAS) is a rare genetic syndrome mostly associated with pathogenic variants in mitochondrial DNA. As there is limited research on the life experience of patients with MELAS, this study aimed to develop an understanding of the patient experience of MELAS through qualitative interviews to identify, describe, and substantiate important and relevant signs, symptoms, and health-related quality-of-life (HRQoL) impact (S/S/I) concepts.

**Methods:**

Clinician and patient interviews were conducted virtually using semi-structured interview guides. During 60-minute interviews with five experts in the United States, clinicians were asked for their perspective on S/S/I of patients with MELAS, patient experience of fatigue and cognitive impairment, and whether patients would be able to accurately report and rate their symptoms and complete a 90-minute patient experience interview. During a 45-minute interview conducted with 16 adults with confirmed pathogenic variant and clinical diagnosis of MELAS, patients were asked about S/S/I. Interviews were recorded, transcribed, anonymized, coded, and analyzed for saturation and concept frequency and clarification (e.g., severity, frequency, duration).

**Results:**

Experts reported 44 distinct S/S and 36 HRQoL impact concepts. All five experts confirmed that cognitive impairment would not inhibit a typical patient’s ability to report on their own experiences; three reported that patients with MELAS would not be able to complete a 90-minute interview. Sixteen patient interviews (mean age: 42.3 [11.1], *n* = 10 women) were conducted. Interviews with patients with MELAS achieved saturation of concept and yielded 35 S/S concepts and 68 HRQoL impacts across 15 domains. The most frequently reported S/S concepts were physical fatigue (*n* = 15, 93.8%), hearing loss (*n* = 13, 81.3%), mental fatigue (*n* = 12, 75.0%), and exercise intolerance and memory problems (*n* = 11, 68.8% each). The most frequently reported impact domains were adaptive behaviors and work impacts (*n* = 14, 87.5% each) and emotional function (*n* = 13, 81.3%).

**Discussion:**

Patients with MELAS can self-report on S/S/I. Results from both patient and clinician interviews demonstrate that symptoms related to fatigue and cognitive impairment are frequent, bothersome, and important to improve. Assessments of fatigue and cognitive function should therefore be considered key outcome measures in clinical trials enrolling patients with MELAS.

**Supplementary Information:**

The online version contains supplementary material available at 10.1186/s41687-025-00962-6.

## Introduction

Mitochondrial encephalomyopathy with lactic acidosis and stroke-like episodes (MELAS) is a rare genetic mitochondrial syndrome that can present in childhood or, more commonly, in adulthood [1, 2]. Although MELAS can be caused by a range of mitochondrial DNA (mtDNA) pathogenic variants, it is most frequently associated with the m.3243 A >G variant in *MT-TL1*, which affects mitochondrial protein synthesis. Because these mtDNA variants negatively impact mitochondrial function, organ systems that have high energy demands (e.g., central nervous system [CNS], skeletal muscle) are most often impacted [3].

Although MELAS symptomatology is broad and heterogenous due to varying degrees of tissue heteroplasmy, the most common symptoms are neurological, and their presence is required to confirm a clinical diagnosis of MELAS. Neurological manifestations can include, but are not limited to, stroke-like episodes, seizures, migraines, and progressive cognitive impairment often leading to dementia [4, 5]. The pathophysiological effects of MELAS, however, are not limited to the CNS and lactic acid increase; muscle weakness, exercise intolerance, and fatigue are also common manifestations. Given that there are no approved or efficacious therapies for MELAS, and current disease management strategies are limited to symptomatic therapies and dietary supplements, individuals with MELAS often experience severe morbidity, poor quality of life, and reduced life expectancy [6].

Patients’ expertise in their own condition is recognized by regulatory bodies as being a key consideration when developing therapies [7, 8]. However, summaries of the MELAS disease experience in the literature have been gathered primarily from clinicians and researchers [1, 2, 9, 10]. Past qualitative interview studies have elicited and summarized the patient experience in a broader grouping of primary mitochondrial diseases (PMD), and this study aims to focus on individuals with MELAS [11–14]. Accordingly, the aims of the current research were to collect expert- and patient-reported data on the MELAS experience, including signs (i.e., observable manifestations), symptoms (i.e., subjectively experienced manifestations), and health-related quality-of-life (HRQoL) impacts.

This non-interventional qualitative study was conducted in two parts. In the first part, clinicians with experience treating patients with MELAS were interviewed. In the second part, following feedback from clinicians that cognitive impairment would not preclude patients with MELAS from participating in qualitative interviews and self-reporting on their MELAS experience, patients with MELAS were interviewed. The primary goal of this study was to develop an understanding of the patient experience of MELAS through qualitative interviews with experts and patients with MELAS for the purposes of identifying, describing, and substantiating the important and relevant signs, symptoms, and HRQoL impacts of MELAS.

## Methods

### Standard protocol approvals, registrations, and patient consents

All patient interview materials were submitted to a centralized institutional review board (IRB), Sterling IRB. No IRB approval was required or sought for the clinician advice meetings. Materials for the conduct of interviews with patients received approval from Sterling IRB on 18 October 2023. All patient interviews were conducted with written and verbal consent, and all clinician advice meetings were conducted with verbal consent.

### Expert advice meetings

Clinicians based in the United States were identified and recruited for expert advice meetings. Five clinicians accepted an invitation to participate in the expert advice meetings. All five clinicians were clinical directors with at least five years of experience with mitochondrial disease and practice at mitochondrial medicine centers that provide extensive multidisciplinary care to patients with PMD [15].

Each advice meeting was conducted via a 60-minute web-based telephone call or teleconference using a semi-structured interview guide and was audio-recorded with each clinician’s verbal consent. During the expert advice meetings, clinicians were asked for their perspective on MELAS signs and symptoms, patient experience of fatigue and cognitive impairment, impacts on daily functioning associated with MELAS, and whether patients with MELAS would be able to (1) accurately report and rate their symptoms through a patient-reported outcome (PRO) assessment with a seven-day recall period and (2) complete a 90-minute patient experience interview. Clinicians were provided the opportunity to spontaneously respond to questions; follow-up questions on fatigue and cognitive impairment were asked only after the expert had been given the opportunity to respond to the open-ended questions. The results of the expert interviews were used to design the structure (i.e., two 45-minute interviews to account for participant fatigue) of the patient interviews. Had experts reported patients with MELAS would have substantial difficulty in participating in interviews or reporting accurately on their symptoms (either due to their levels of fatigue or cognitive impairment), consideration would have been given to the conduct of dyad patient/caregiver interviews or direct caregiver interviews.

### Patient interviews

Conduct of two 45-minute interviews each with approximately 15 adult patients with MELAS were planned for this study. The target of 15 interviews was based on the rarity of the disease and an estimate that 15–20 interviews are expected to be sufficient for demonstrating saturation [16, 17]. The first interview was intended to spontaneously elicit patient perspectives about the experience of MELAS. During the second 45-minute interview, select measures of fatigue and cognitive function were cognitively debriefed with the participants; these results are outside the scope of this paper.

Recruitment of patients with MELAS was conducted via clinicians and patient advocacy groups who were asked to advertise and/or identify potentially eligible participants with a confirmed diagnosis of MELAS to participate in interviews.

Interviews with participants were conducted via telephone or web-based teleconference. Eligible participants needed to meet the following criteria:

- 18 years of age or older.
- Genetic confirmation of a MELAS-associated pathogenic variant and clinical manifestations consistent with a MELAS clinical diagnosis.
  - A Clinician Diagnosis Confirmation Form, completed by the participant’s physician, confirmed a medical history with at least two of the following: seizures with neurological deficits, recurrent headaches, encephalopathy, and/or magnetic resonance imaging (MRI)/‌electroencephalogram (EEG) findings consistent with MELAS.
- Provided written informed consent to participate in an interview by completing the Informed Consent Form and Health Insurance Portability and Accountability Act (HIPAA) Authorization.
- Fluent in English (i.e., able to speak, read, write, and comprehend) and willing and able to participate in two audio-recorded, 45-minute, web-based teleconference or telephone interviews.

Participants were excluded if they had a condition or situation that could interfere with their participation in the study (e.g., severe cognitive impairment or hearing impairment) to an extent a caregiver would be required.

Before starting the interviews, the interviewers participated in a mock interview to ensure consistency in data collection and to encourage interviewing best practices. During participant interviews, interviewers followed a semi-structured interview guide to ask a series of open-ended questions designed to elicit spontaneous descriptions of the signs, symptoms, and HRQoL impacts of MELAS from the patient perspective. Participants were asked about their most bothersome sign/symptoms and HRQoL impacts, as well as the sign/symptoms and HRQoL impacts that were most important to improve. Targeted probes were used to obtain specific information on concepts of interest (i.e., cognitive impairment and fatigue) only after the participant had been given the opportunity to respond to the open-ended questions spontaneously. Concepts that were reported as probed were not included in the saturation analysis. While no dyad interviews were anticipated during the interview planning, there were two instances in which participants’ caregivers remained and contributed to the patient interviews. In these instances, concepts that were first reported by participants were treated normally, while concepts that were reported by caregivers were treated as probed upon receiving confirmation from the patient that the concept was relevant to their experience.

Each interview was audio recorded with each study participant’s written and verbal consent.

### Expert and patient interview transcription and coding

Audio recordings of the interviews were sent to and transcribed by an independent transcription company. Transcripts were anonymized by removing any potentially identifying information (e.g., names of people, medical centers, or places) from the transcript. The anonymized transcripts served as the source data for analysis.

Transcripts were content analyzed utilizing published qualitative approaches and recommendations from Patrick et al. (2011) Parts 1 and 2 and others [18–21]. Both expert advice meeting and patients interview transcripts were coded based on a preliminary codebook generated from each respective interview guide that included placeholder codes for types of information expected to be elicited. As concepts emerged from the advice meeting or patient interviews, new codes were created. Three researchers were involved in the coding process. Coding was conducted in ATLAS.ti version 9 software (ATLAS.ti Scientific Software Development GmbH, Berlin, Germany) [22, 23]. During the coding process, the coders communicated each time there was a modification to the codebook or if issues arose in an effort to maintain a consistent use (or addition) of codes. After all transcripts were coded, a harmonization meeting was held to ensure consistency of coding across all transcripts and coders. Unused codes were removed. Codes deemed to be evaluating the same aspects were merged. Following harmonization, the coded transcripts were used to develop a set of findings tables, including verbatim patient quotes, to inform the conduct of qualitative data analysis.

### Analysis of expert advice meeting data

Expert data were collected and analyzed with respect to clinicians’ specialty, years of experience treating patients with MELAS, the setting and size of their current practice, prior clinical trial experience specific to MELAS, and the number of patients with MELAS seen per month. Data were also collected on concept frequency and clarification; concept frequency was determined by counting the number of unique experts who reported the concept at least once during an advice meeting. Concept clarification was assessed based on the salient aspects of a concept (e.g., severity, frequency, duration, or other characteristics).

### Analysis of patient interview data

An interim analysis of the first eight interviews was conducted to determine the sign, symptoms, and impacts reported to date and to report on any issues associated with interview conduct. No changes to the interview guide were made due to the results of the interim analysis. Patient data were formally analyzed for saturation, concept frequency, and concept clarification. Qualitative data were assessed for conceptual saturation to confirm the adequacy of the sample size [24] to draw conclusions about the target patient population. Saturation was analyzed at the concept level for symptoms (e.g., headache) and the domain level (e.g., social impact) for impacts. Concepts that were reported spontaneously by participants were analyzed in sequential groups, in chronological order of occurrence. No new concepts emerging in the final transcript group was an indicator that saturation of the concept had been achieved.

Concept frequency refers to the number of participants with MELAS who expressed a given concept (sign, symptom, or impact) during the CE interviews. Though signs and symptoms are differentiated by the fact that signs can be objectively observed and symptoms can only be felt by the patient, signs may also be reported and described by the patient in some cases [25]. For the purpose of reporting, signs and symptoms were grouped together because an emphasis was placed on understanding the experience of MELAS as described and reported by patients. The participant concept frequency count was determined by totaling the number of unique participants who mentioned during the interviews that they experienced a concept at least once during their experience with MELAS. Concept clarification analyses were conducted to better understand what participants with MELAS consider to be the most salient features, or dimensions, of each sign, symptom, or HRQoL impact concept elicited (e.g., severity, frequency, duration). To clarify the important dimensions of each elicited concept, participant quotes were coded with respect to their most salient aspects [20].

## Results

### Expert advice meeting results

#### Expert advice meeting – expert characteristics

Interview data from all five experts were included in the analysis. Experts’ years of experience with mitochondrial diseases ranged from 8 to 20 years. All five experts worked at different academic medical centers, with the size of their practices being between 5 and 12 medical staff. Experts estimated working with between 2 and 40 adult patients with MELAS per month, and all five reported having experience with MELAS-specific clinical trials.

#### Expert advice meeting – Signs and symptoms

Experts reported 44 distinct MELAS signs and symptoms, all of which were reported spontaneously by at least one expert, without the need for a probe. Physical fatigue, seizures, stroke-like episodes, exercise intolerance, memory loss, mental fatigue, impaired executive function, cardiac involvement, diabetes, hearing loss, migraine, and impaired visuospatial abilities were reported by all five (100%) experts. Difficulty concentrating, gastrointestinal dysmotility, headaches, dementia, vision impairment, and weakness were reported by four of the experts (80%). The symptoms considered most concerning to experts were physical fatigue (*n* = 3), memory loss (*n* = 3), stroke-like episodes (*n* = 2), and exercise intolerance (*n* = 2). For a complete list of expert-reported MELAS signs and symptoms, including descriptions, frequencies, and level of concern, please see Appendix A.

#### Expert advice meeting – Impacts

Experts reported a total of 36 MELAS-related impact concepts that were organized into 14 domains. The impact domains reported by the most clinicians were emotional function, independence, and work/school (all reported by *n* = 5, 100%). The most frequently reported impact concepts were requiring help from others (*n* = 5, 100%), decreased performance in work/school (*n* = 4, 80%), and impacted sleep (*n* = 4, 80%). For a complete list of expert-reported impacts, please see Appendix A.

#### Expert advice meeting – Patients’ ability to self-report on their experience with MELAS

Experts reported on patients with MELAS cognitive impairment and their ability to complete a potential 90-minute patient-experience interview. Experts reported that whether patients with MELAS were aware of their cognitive impairment was dependent on the patient and the severity of their condition. All five experts (100%) confirmed that for most patients with MELAS, cognitive impairments would not inhibit their ability to report on their own experience with MELAS through a PRO assessment with a seven-day recall period. However, in the smaller subset of patients with more severe disease, a PRO assessment would not be appropriate.

Three experts (60%) reported that patients with MELAS would not be able to complete a 90-minute interview and recommended conducting interviews in shorter intervals.

### Patient interview results

Due to the nature of MELAS-related fatigue and cognitive impairments, interviews tended to run longer than anticipated and lasted between 45 min and 60 min. In addition, two participants unexpectedly had a caregiver present at the time of their interviews, and their data were retained. This decision was made based on the rationale that the participants could spontaneously report on their experiences, thus providing key insights into their MELAS diagnosis; the caregivers provided additional details on concepts as necessary.

#### Patient interviews – Patient demographic data

The findings of the expert advice meetings informed the conduct of the patient interviews. Sixteen adult participants with clinician-confirmed diagnoses of MELAS participated in the interviews. Patient characteristics are summarized in Table 1.

**Table 1:** Patient demographic and health information table

Characteristic	Participants (*N* = 16) *n* (%)
Age	
Range in years (min–max)	24–61
Mean (standard deviation)	42.3 (11.1)
Country	
US	15 (93.8%)
Canada	1 (6.3%)
Sex assigned at birth	
Female	10 (62.5%)
Male	6 (37.5%)
Gender identity	
Woman	10 (62.5%)
Man	6 (37.5%)
Hispanic, Latino/a/x, Spanish origin	
Not of Hispanic, Latino/a/x, or Spanish origin	16 (100%)
Race	
Asian	1 (6.3%)
Black or African American	1 (6.3%)
White	14 (87.5%)
Highest level of education	
Some college or certificate program	2 (12.5%)
Completed certificate program or associate degree	1 (6.3%)
Undergraduate degree	6 (37.5%)
Post-graduate degree	6 (37.5%)
Not provided	1 (6.3%)
Current work status	
Working full-time	3 (18.8%)
Working part-time	1 (6.3%)
Student	1 (6.3%)
Not currently working due to MELAS	8 (50.0%)
Retired	1 (6.3%)
Unemployed	2 (12.5%)
Overall health	
Very good	2 (12.5%)
Good	7 (43.8%)
Fair	5 (31.3%)
Poor	2 (12.5%)
Age of onset (in years)	
0–9	3 (18.8%)
10–19	3 (18.8%)
20–29	4 (25.0%)
30–39	3 (18.8%)
40–49	3 (18.8%)
MELAS genetic diagnosis/pathogenic variant	
m.3243 A > G	16 (100%)
MELAS Clinician Diagnosis Confirmation Form subcategories (history of ≥ 2 required for eligibility)	
MRI and/or EEG findings consistent with MELAS	11 (68.8%)
Recurrent headaches, sometimes associated with vomiting	10 (62.5%)
Seizures (focal or generalized) often accompanied by sudden focal neurological deficits	9 (56.3%)
Encephalopathy with altered level of consciousness with or without acute mental deterioration, which can be slowly progressive and/or reversible	3 (18.8%)

#### Patient interviews – Saturation

No new symptom concepts or impact domains emerged (i.e., were reported for the first time) in the final group of four interviews, indicating saturation of concept was achieved and confirming the adequacy of the sample size. Twenty-nine symptom concepts were spontaneously reported by participants. A total of 15 impact domains were spontaneously reported by participants in relation to their experience of MELAS. Of these impact domains, all were reported prior to the final group of interviews. Please refer to Appendix B for the saturation frequency graphs, which show a running total of new symptom concepts or impact domains reported after each participant interview.

#### Patient interviews – Signs and symptoms

Including both spontaneous and probed concepts, participants reported a total of 35 signs and symptoms. The most frequently reported symptoms were physical fatigue (*n* = 15, 93.8%), hearing loss (*n* = 13, 81.3%), mental fatigue (*n* = 12, 75.0%), exercise intolerance (*n* = 11, 68.8%), and memory problems (*n* = 11, 68.8%).

The symptoms reported as most bothersome by two or more participants were physical fatigue (*n* = 6), migraines/headaches (*n* = 5), hearing loss (*n* = 2), difficulty finding words/expressing speech (*n* = 2), and difficulty comprehending speech (*n* = 2). Nine participants reported physical fatigue as the most important symptom to improve, followed by weakness (*n* = 2), migraines/headaches (*n* = 2), and difficulty finding words/expressing speech (*n* = 2). The degree to which symptoms like fatigue and cognitive impairment affected participants can be seen through the following patient quotes:

- **Participant 01–07**: “Fatigue is more than just being tired. It’s kind of like hitting a wall energy wise. And just not being able to follow through. It’s almost like you could stop mid-task and fall asleep of what you were doing. And it’s just – it’s more than exhaustion. It’s more than just a little sleepy. It’s, it’s like you, you’ve hit a wall. I don’t know how else to describe it.”
- **Participant 01–28**: “Um, I get really tired after doing – running errands or any kind of that. I’m pretty much always tired.… Um, usually when I get back home from like going to the grocery store, I’m just like, I’m just tired of walking around I guess.… It’s not like sleepiness tired, but it’s like need to sit down exhausted kind of thing. One usually flows into the other.… Um, even like walking around the block for I guess exercise. That’s exercise for me, it’s just one block. Um, I will get extra, extra tired and I will get a migraine and lie down for the rest of the day.… I think exercising, doing the same amount of exercise as someone without MELAS is extra exhausting for me.… Like running a mile for me is different than running a mile for everyone else. Like I feel more exhausted than everyone else might be.”
- **Participant 01–02**: “I was in the hospital, um, the last time I was in the hospital, they finally, um, diagnosed me with MELAS.… They’d say, yeah, but what’s the name of the hospital. I couldn’t give them the name of the hospital. They would ask me who the president was. I couldn’t remember, um, his name, but I could remember specifics about him. I couldn’t tell time. Um, and so it was, it was really bad.… Um, I do, um, um, lose words a lot, a lot. Um, and so Google and I have gotten very comfortable because I’ll be maybe trying to write something and I know the word that I want to use and I can’t remember the word or, um, if I can remember the word but I can’t remember how to spell the word or how to say it.”

For concept descriptions and frequencies of all concepts reported by more than one participant, please see Table 2. For additional exemplary participant quotes, see Table 3. For a version of the table with all concepts, including those reported by only one participant, please see Appendix C.

**Table 2:** Patient-reported sign and symptom concept description table (*n* > 1)

MELAS-related sign/symptom^*^	Concept description^†^	Frequency of participant reports (*N* = 16)^‡^ *n* (%)	Reported as most bothersome (*N* = 16)^§^ *n*	Reported as most important symptom to improve (*N* = 16)^**^ *n*
Physical fatigue	Participants described being lethargic, tired, and lacking energy to complete tasks (e.g., household chores, exercise, social activities) and/or needing to nap and take breaks	15 (93.8%)	6	9
Hearing loss	Participants described needing hearing aids or cochlear implants due to being hard of hearing or not being able to hear at all	13 (81.3%)	2	1
Mental fatigue	Participants described mental fatigue as needing to conserve energy for mental tasks, feeling depleted or tired, and not being able to complete tasks, inclusive of speaking to others and concentrating, or focus on them	12 (75.0%)	0	0
Exercise intolerance	Participants described a decrease in exercise performance, not having the energy to exercise or keep up with their peers, reduced stamina, needing an increased rest following exercise, and not being able to exercise due to fatigue; participants described exercise as walking, jogging, running, lifting, rock climbing, biking, and cleaning	11 (68.8%)	0	1
Memory problems	Participants described memory problems as short-term or permanent memory loss wherein they cannot remember names, words, schedules, appointments, etc., and experience overall poorer memory	11 (68.8%)	0	1
Weakness	Participants described decreased performance, myopathy, and not being able to perform routine tasks without soreness, as well as feeling tired or slow in various areas of their body, such as their hands or feet, from regular or strenuous activity	10 (62.5%)	1	2
Difficulty concentrating	Participants described having difficulty completing only one task, needing to stop and think about tasks performed, easily forgetting completed tasks, and taking longer to or being unable to keep up with group conversations	9 (56.3%)	0	0
Migraines/‌headaches	Participants described needing to take medication to deal with migraines, as the migraines can vary in severity anywhere between a dull pain and the feeling that one’s brain is burning or that there is a jackhammer being taken to one’s head. Participants reported that the migraines can cause nausea and feel like a stabbing or pressure sensation that may occur along with auras. Participants reported that the migraines may be related to seizures and that they need to sleep to deal with the severity of the migraines. For their experience of headaches, participants described headaches occurring around the head and neck area. If participants experienced both headaches and migraines, they noted that migraines were more severe than headaches.	9 (56.3%)	5	2
Muscle fatigue	Participants describe muscle fatigue and inability to move and/or lift weights, and muscles not performing as expected	9 (56.3%)	0	0
Difficulty finding words/expressing speech	Participants described having difficulty finding the words to convey and express their thoughts; participants described impaired ability to have conversations and resulting feelings of frustration	7 (43.8%)	2	2
Seizures	Participants described seizures as making their speech slow and slurred, immobilizing them, losing cognition, and being mild to massively severe. Participants described needing to be hospitalized due to seizures, and that the seizures may be caused by a variety of reasons (e.g., lack of sleep, missing medicines, multiple sounds).	7 (43.8%)	0	1
Strokes and stroke-like episodes	Participants describe stroke-like episodes and strokes as having leg weakness, brain blurriness, tunnel vision, impaired communication, and not being able to walk or speak. Participants described needing to be hospitalized due to stroke-like episodes.	7 (43.8%)	1	1
Balance issues	Participants described balance issues as leaning or tilting to the side, wobbling when standing, and losing one’s balance without the support of a cane, bed, or chair	6 (37.5%)	0	0
MELAS-related symptoms of diabetes	Participants described having symptoms of diabetes and insulin-resistance due to MELAS and needing to take insulin and eat a restricted diet	6 (37.5%)	1	0
Vision impairment	Participants described having a loss of vision in their central vision, near- and farsightedness, double vision, progressive vision loss, and differences in sight ability for each eye. Participants described not being able to see other peoples’ physical features and needing glasses/other vision aids.	5 (31.3%)	1	0
Cardiac involvement	Participants described experiencing tachycardia, atrial fibrillation, and high heart rate because of MELAS	3 (18.8%)	0	0
MELAS-related gastrointestinal issues	Participants described experiencing constipation, slow bowel motility, and overall digestive issues	3 (18.8%)	0	0
Pain	Participants described pain and burning as occurring in the muscles and abdomen that may or may not occur following activity, which does not resolve based on massage or stretching, or due to the need to have a bowel movement	3 (18.8%)	0	0
Brain fog	Participants described brain fog as fuzzy thinking, difficulty concentrating, and having difficulty expressing themselves, as well as not being able to wake up and falling back asleep constantly, as well as spacing out and forgetting things	2 (12.5%)	1	1
Difficulty comprehending speech	Participants described cognitive-related issues with understanding, processing, and explaining speech when speaking or writing	2 (12.5%)	2	1
Difficulty processing	Participants described difficulty processing as having difficulty communicating when participating in conversation	2 (12.5%)	0	0
Difficulty reading	Participants described a significant decrease in reading level that occurred after a stroke or experiencing notable mental fatigue when reading	2 (12.5%)	0	0
Muscle numbness	Participants described muscle numbness as a tingling feeling or a numb feeling	2 (12.5%)	0	0
Tinnitus	Participants described experiencing ringing in their ears or a constant “eee” sound in their hearing	2 (12.5%)	0	0

^*^Concept reported by study participant

^†^Description of concept based on study participant report

^‡^Frequency is presented as the total number and percentage of study participants who reported each concept at least once

^§^Most bothersome symptom as reported by the study participant, percentages not provided as participants were permitted to report on more than one symptom as most bothersome

^**^Most important symptom to improve as reported by the study participant, percentages not provided as participants were permitted to report on more than one symptom as most bothersome

**Table 3:** Exemplary quotes representing most common sign and symptom concepts^* *^Defined as reported by ≥ 7 participants

Concept	Exemplary quotation
Physical fatigue (*n* = 15)	**01–01** “Um, it means I don’t get to do the things I normally would want to do because I’m too tired and too lethargic. I don’t have, I don’t have the energy to do them. Like take a shower, do the dishes, cook, clean.… Like literally all of my waking hours, I’m fatigued. I don’t have the energy that I need to.… Um, no. I just don’t have the energy to go exercise. If I want to do the dishes or if I need to go to an appointment or go do the grocery shopping or an errand, then I don’t get to exercise because my energy was put towards those other items.” **01–23** “I get the feeling like you’re lethargic and not – and I get – my speech will get a little bit slurred and not able to complete things around the house that I normally would be able to do. It would be more of a day where I possibly wouldn’t be able to leave the bedroom for the day or if I went from the bedroom to the living room, I wouldn’t be able to really do anything. I would, um, would need my husband to do things for me that say like make a coffee for me or make food for me that I’m not – wouldn’t be normally completely able to do.… It’s throughout half the day like the fatigue is where I end up sleeping much longer than I normally would or get up from the bed much longer than I usually do and it’s not – yeah. I don’t have trouble, trouble sleeping. It’s more, you know, I’m so – my body is so exhausted, I can’t get up.” **01–24** “Um, sometimes when I’m not exercising and just like in normal life, I do have I think more tiredness than other people, but it’s not the same level of exhaustion. It’s just really like more that I can’t – hmm, I think normal people like – probably they wake up and they like feel energized if you sleep eight hours. That’s not always the case for me. Sometimes I wake up and I’m like still kind of like draggy tired. And then I like might sometimes in the afternoon I’ll get tired, you know, have like a wave of being tired for like two hours.”
Hearing loss (*n* = 13)	**01–02** “I have two hearing aids…. PARTICIPANT: Um, you said what now about the hearing? INTERVIEWER: Can you tell me a bit about your hearing loss? PARTICIPANT: Oh, yes. Um, I have two hearing aids. Um, and, um, I have, um, good technology on my, my phone where I can keep up with, but it’s very, very hard, um, having MELAS and having hearing aids because it gets really hard when you are trying to have conversations with people and you don’t understand anything that the person is saying, and so you already have comprehension issues and you have hearing issues, so it’s very hard to have conversations with people.” **01–04** “Um, early on when I was first diagnosed, um, I started to have hearing loss, um, shortly followed by, uh, exercise intolerance.… Um, my ex-wife in hindsight feels that there was some level of hearing loss in my early twenties. Um, so certainly there were, you know, times, say like Christmas, Thanksgiving dinner, big table, you know, there were, there were issues in my mid to late twenties where just I wasn’t excluded from conversation but I, I wasn’t a part of it ‘cause I just – big table, lots of noise, couldn’t discriminate conversations.” **01–07** “I have a mild to moderate to severe deafness on my audiogram. I use two hearing aids. Mostly I read lips. So I can’t see your face, so, uh, it’s a little harder.”
Mental fatigue (*n* = 12)	**01–05** “And the way I see mental fatigue is, uh, difficulty more than usual of understanding speech. So if, if I’m tired… it takes a lot of brainpower to process the speech different than, uh, hearing through ears. And that’s where I notice the fatigue. And that’s maybe – it, it depends on the environment. If, you know, I’m in a social setting….I’ll see that, you know, after an hour or so or I’ll see that and whatever. **01–15** “Um, you kind of end up in a daze. You could mindlessly scroll through the internet and accomplish nothing and realize that’s been three hours.” **01–28** “Uh, you just feel like I’m not doing anything productive. Like I know I have like easy tasks that I could be doing and I just don’t feel motivated. I guess I just don’t – like I’ll stare at it and just not I guess – or I zone out a lot I guess.”
Exercise intolerance (*n* = 11)	**01–05** “My activities like sitting down talking to you, you would never seem fatigued, but I get up, I do some exercise – when I say some exercise, uh, I was doing some cleaning or whatever. I would say my activity is cleaning the countertops or whatever. It’s like, yeah, I have 20 minutes. I need to sit down and take a break…. Uh, generally I’m – it’s like I’m just unable to move any farther. I struggle to take another step or whatever.” **01–23** “I cannot walk a long distance with getting – feeling really exhausted. I have physical therapy for an hour long and then my – I’m completely, energy is spent. My whole body feels completely wiped out. Um, if I were to go to a gym and walk on the treadmill for a little bit or just a little bit of cardio, I would – I get completely like again, like my whole body is spent for the rest of the day.… My complete – my battery is depleted.” **01–25** “Um, so basically I have to change my workouts to kind of suit my energy levels.… So I used to do a lot of really high intensity workouts, um, and I was a huge runner. So I used to go and like run three to four miles a day and, um, do a lot of like circuit training and basically like kind of like cross fit stuff. Um, and recently – well not recently. I guess I’d probably say in the past year and a half, two years I’ve had to like really adjust that, um, and do more low intensity workouts. Also things like Pilates, um, just like slower things that don’t really burn my energy as fast as the other workouts would have.”
Memory problems (*n* = 11)	**01–04** “Um, but I, I guess, guess it was the word-finding issues and the – some of the short-term memory stuff. Um, you know, my ex felt like there were times like that it wasn’t the hearing loss where we had to go over things multiple times as far as kids’ schedule, stuff like that.” **01–20** “And then other times I forget names, uh, um, and I know that, um, it becomes very frustrating and there are some things – uh, people who are famous or people who I have known, um, and those kinds of things. And those kind of things can, um, just come up sometimes and, I don’t know, the next day I remember those things. So, um, and, um, I think that the thing I can just say is that, um, if I’m writing on a subject, um, I will forget a word and I can’t even think of a, um – I know what the word is supposed to mean but I, I can’t even think of, um, a different way to say the same thing.” **01–23** “Those are definitely – you know, my memory has gotten a little bit worse. Takes longer for me to think of, think of things and remember things. Luckily, you know, it hasn’t got – yeah, in the past year or so, I don’t know if it’s because, you know, I’m getting older or, you know, it is MELAS but, but I think, you know, it is triggered by MELAS, the forgetting things, the cognitive – it’s not super bad yet, but, you know, my memory has gotten a little – gotten weird.… Like it takes longer for me to remember things or think of things. Like say, you know, think of like a certain actor or certain like, you know, memory, remember a certain thing that happened. And then it will take me say like a day to, you know, remember the fact or remember certain things”
Weakness (*n* = 10)	**01–05** “Um, if I’m doing any exercises with my arms….three or sometimes a five pound weight that I’m able to do some exercises with. So if I have to lift something over my head that ….Yeah. If, um, you wanted to look at it, um, I’m not able to run. Uh, if I went and tried to jump vertically, I’m barely able to clear my feet off the ground….That’s where it is. As opposed to in my twenties….Yes. Um, in my thirties, I didn’t have much muscle weakness. Uh, in the past five years, it’s really drastically decreased…. Um, I’d consider it muscle deterioration, like basically it’s a weakness. That’s what, at least that’s the way I see it. **01–15** “That, uh, it, it feels like I have completed a very intense workout when I haven’t. You know, I’ve literally carried around a, an iPad for an hour or two and suddenly my arms are exhausted, in a lot of pain. It’s pulled my shoulders. It’s – and it’s just an iPad. Like that’s not heavy. Um, and, uh, weakness, I’ve, I’ve actually run into a situation where, uh, at my previous job I, I used to do some sweeping and I did it very leisurely like one small section every hour, um, for an eight-hour day and then the next day my arms were not responding. I couldn’t make them move. They felt deadweight, no feeling. They didn’t even hurt at that point. They were just weak and non-responsive. That was terrifying.…” **01–23** “I have, um, very weak muscles throughout my body and they have gotten weakened. Maybe started weakening about 25 years ago. And then I’ll do like – it’s progressively gotten bad. It’s affecting – for the last ten years my balance has progressively gotten really bad because my muscles are so poor throughout my body.”
Difficulty concentrating (*n* = 9)	**01–04** “I guess I’ll say with paperwork for work. You know, my computer at home and I’ll get three-quarters of the way through one note and then realize I have I don’t know, some other thought that goes through my mind and I’ve got to, you know, write that down on a post-it note or, um, next thing, you know, like, like, oh I, I need a snack, you know. Um, I feel like my mind drifts a bit, you know, and I can’t – there’s times I’m, I’m not completing one task just to go do something else shortly and then come back to it. Um, I think that kind of drives me to be doing three things, four things at once.” **01–20** “I feel like I, uh, I have a more limited, um, um, time for how long I can stay focused on a project.… I think that those are, those are the times when, um, when I just, um, uh, don’t focus on anything. Um, so from, uh – let’s say it can be so much of an impediment that I can’t read a line of a book.… I, um, before I got, um MELAS, I, um, I had to – uh, I got a Bachelor’s degree and two Master degrees in, um, to study things like that…. All of that needed me to stay focused on a topic and would take a long time. Um, and I, um, I feel like, um, to do, um – it does mean that I think I have just a, a shorter time I can stay focused on one topic and I need to, um – I can stay active, but I will need to change what activity I’m doing.” **01–24** “Like I, um, work for myself, so I will sit down to do like a task and I get very easily distracted. So it’ll be like three hours later and I still haven’t done something that should take me like 20 minutes because I just kind of totally lost track of time and, um, started doing something else.”
Migraines/‌headaches (*n* = 9)	**01–06** “But, um, these headaches – they turn into headaches. Usually they happen at night. Or like sometimes they happen if I do too much. Like if I go to a concert or if I walk too much or if I’ve been like at Disneyworld all day or something like that…. Um, they turn into headaches at night and then they turn into migraines if I don’t do anything about it. Um, I’ve tried….migraine pads and Advil…. I just go to sleep…. Eventually your battery runs out and you’re just like, you want to do stuff but you can’t and then you just do it the next day because you have no energy.…” **01–23** “I still get chronic migraines. Um, I also now, you know, continue to have the chronic migraines.… It feels like a hammer is attacking. I get the, um, the aura along with it, sensitive to noise and light and it happens with both sides of my head. It switches different, you know, right or left. I have to put an icepack on my head for it to help, um, the throbbing, the hammering. There’s like a jackhammer in my head. And then there’s also like feels sometimes like a pinball is happening in my head and bouncing around.… And nausea.” **01–25** “Um, so I get them behind my left eye. It’s usually on my left side that I feel it. It kind of goes behind my left eye to my ear and then down the back of my neck, which is like – that’s like a really bad one. Usually it kind of just sits behind my eye. Um, it kind of just feels like this like – I want to say like dull pain, but like it’s also stabbing. It’s like stabbing behind the eye and then – but it’s like lasting and it lasts so long and it makes you feel so nauseous and I get really sensitive to light as well. That’s like one of my biggest symptoms. So whenever I have a migraine, like the only thing I want to do is like close my eyes because the light hurts.… But it takes – probably the longest migraine I’ve had has been, uh, probably two days, 45 hours.”
Muscle fatigue (*n* = 9)	**01–05** “Muscle fatigue is I’m walking [INDISCERNIBLE] down the sidewalk or whatever and we’re walking and I have to stop and pause because my – I’m feeling my legs are having difficulty, uh, continuing to walk. And if I tried to push it, I might risk a fall.” **01–25** “Um, so I do get pretty bad muscle fatigue after I do any type of lifting. Um, and I watch it ‘cause I know that we have like the issues with lactic acid. So I’ve never had like severe pain, but I definitely take a lot longer to recover than I guess a normal person….so if I do have a lift, it takes several days before I can even do another activity that’s like that.” **01–28** “Um, just carrying in groceries and stuff, that makes me really – just makes me feel weak or tired, like muscle weakness after carrying anything. Um, sometimes when I wake up, it’s hard to get yourself moving or sometimes I get random – my leg doesn’t want to move or just random sometimes.…”
Difficulty finding words/‌expressing speech (*n* = 7)	**01–02** “Um, I do, um, um, lose words a lot, a lot. Um, and so Google and I have gotten very comfortable because I’ll be maybe trying to write something and I know the word that I want to use and I can’t remember the word or, um, if I can remember the word but I can’t remember how to spell the word or how to say it. So Google and I have gotten very, very comfortable with each other. And, um, yeah, there’s a lot, a lot of times where I lose, lose words. And, um, it was embarrassing at first, but now I just make myself look like I’m thinking about it and I’m, you know, being smart. But I’m really – I can’t remember what, what the word is.” **01–20** “So I do forget words that I want to say, and that happens, um, when I’m experiencing – not just – I am much more speech impediment when I had the seizure, but I still forget words, um, that I want to say when I want to write.… my speech capability. It may – it’s probably, um, less than, um, before I got diagnosed with MELAS I think.… But as I mentioned, I do sometimes forget words. That becomes frustrating. Um, and it does allow me to take an additional amount of time to think, uh, and try to recover – remember words and things like that.” **01–22** “Um, I know what, um, what word I want to say, but it won’t come out straight. Like it, it just, um – I can’t talk straight. Um, and it’s getting worse now, but it’s not – so it’s hard to have a conversation because, um, um, I can’t talk, um, straight and it’s, um, it’s really hard.… Because I know what I want to say in my brain, but it comes out either backwards or, um – CAREGIVER: You really can’t find the word. PARTICIPANT: Yeah. I can’t find the word to like – I know what I want to say, but it’s really – it won’t come out.” -
Seizures (*n* = 7)	**01–02** “it’s gotten to the point now where I may have an episode. And episodes, um, make me very, very tired after I have the episode. And this time I was very, very tired after the episode, but then all of the sudden my speech was very slurry and slow and so I told my husband, I said, okay, now we need to go to the, um, emergency room. And so yeah, I was at the emergency room, um – that was just – that was not too long ago.” **01–16** “Uh, when I had my seizures, uh, the first one….my wife, uh, saw that I was, uh, uh, sleeping, uh – that I wasn’t, uh, communicating. I was fully asleep, like I wasn’t moving, uh, versus, uh, what you would end up seeing in many of the seizures, like, uh, on, on TV. Uh, so it was more I was, uh, I wasn’t moving.… Uh, the only – uh, I know I’ve had multiple seizures where I’m, uh – I was, uh – my body was, uh, doing the exact thing that you would end up seeing on like TVs or, uh, YouTube in which it’s like you’re convulsing. Yeah. Uh, and so, uh, I’ve had I’d say two where I’m like falling – like where I’m in more of a sleep, uh, and then three of them where – well two of them I was in the hospital and I was, uh – my body was, uh, uh, having more of like convulsions” **01–23** “Um, with – um, actually I have been diagnosed with epilepsy because I have tiny, little second-long little seizures. So I guess that would be seizure-like.… Um, yeah. But the longer ones, that wasn’t like a seizure, but it was – I, I call it falling on your face because I broke my, um – I fell on my face and I needed stitches. And my lip broke some teeth and – because I was wearing my glasses, it cut into my nose. But the episode is a feeling of like, um, complete numbness throughout your body that you can’t control. It feels, um, like your head is heavy. Um, and then there’s also – I get this like – in my head, it feels like someone is trying to like tap my brain. I know it’s so weird of a sensation, sensation.” -
Strokes and stroke-like episodes (*n* = 7)	**01–16** “Again, in… uh, 2022, uh, I had a stroke. And, uh, so I was in the hospital for probably two weeks.… Uh, after I had my stroke, uh, I’m, uh, still struggling with my speech. Uh, and remembering everything that did happen. Uh, it’s definitely improving over time, but once I had the stroke then, uh, in 2022, uh, that’s when, uh, I almost forgot, uh, people’s names.… But the stroke was the biggest issue in terms of my speech, my, uh – what they call OT for my hands. Uh, again I’m not perfect. Like basically everything since MELAS, uh, it all got worse and worse. But, uh, the stroke was the most painful, uh, thing that, uh, my body just couldn’t connect.” **01–20** “I first got diagnosed when I experienced a stroke. Um, and I had tunnel vision. I lost the ability to communicate by any means and I lost the ability to write letters of the alphabet or draw. I just could not communicate.” **01–27** “And then, um, I’ve had three, uh, stroke-like symptoms, uh, over the past like five years and that’s really, um, taken out of me.… I, um – my first major one was, uh, uh, 2019 and I was in the hospital for 22 days. And, um, that was when – so after my fire season and stuff like that, I had a really stressful, uh, fire season. I had got off and then like it was right after the holidays, um, my wife noticed that I wasn’t really being myself and stuff. So I, um, then, uh, I was on the couch and I told her that something was wrong. And, uh, she took me to the hospital and that’s when I was in the hospital for so long and they couldn’t figure out what was wrong with me…They did all kinds of testing and it took three years and two stroke-like incidents to finally figure out that that’s when I got diagnosed with, um, uh, MELAS. And then right after that, I had a very minor stroke-like episode…. Um, just like, uh, pretty much a regular stroke. Like, uh, stroke symptoms.… And, um, like tingling on my side. Um, just like all of the sudden, um, my whole left side went numb and, uh, uh, like the second one – like the first one I didn’t even know what was going on. Like I ended up like the first two weeks I don’t remember anything, but the second one it was definitely the – like a stroke. Like I had my whole left side went numb. I couldn’t open my left eye, um, couldn’t move my limbs or anything and it was extremely like a stroke. And then the third one was kind of like that, but it only lasted like 15–20 minutes and then I pretty much snapped out of it and everything was fine. It just, it kicked my butt really bad for a week, but, uh, that was the most like minor one I’ve had.”

#### Patient interviews – impacts

Sixty-eight HRQoL impact concepts emerged in 15 impact domains. The impact domains reported by the most participants were adaptive behaviors (*n* = 14, 87.5%), which included the need for hearing aids/cochlear implants (*n* = 10, 62.5%) and impacted eating habits (*n* = 3, 18.8%); work impacts (*n* = 14, 87.5%), which included being unable to work (*n* = 8, 50.0%) and needing to change career paths (*n* = 4, 25.0%); and emotional function (*n* = 13, 81.3%), which included feeling frustrated (*n* = 4, 25.0%) and feeling anxious (*n* = 3, 18.8%) and/or depressed (*n* = 3, 18.8%). The most frequently reported specific impact concepts were the need for hearing aids and cochlear implants (*n* = 10, 62.5%; adaptive behaviors domain), inability to work (*n* = 8, 50.0%; work domain), and impacted family and social relationships (*n* = 7, 43.8% family/friend relationship domain). Participants often described their most bothersome impacts in terms of symptoms (e.g., impacts associated with cognitive impairment or fatigue).

- **Participant 01–02**: “Um, and so sometimes I have to have my husband take – go with me because I need an, um, uh, uh, advocate, you know, who can actually hear what has been said because sometimes I don’t understand it. Um, so it’s hard having two hearing aids too, so.”
- **Participant 01–01**: “Well I feel like if you are fatigued, in general if a person is, is fatigued like too much, that impedes your ability to participate in what you want to do. And that, that’s the case for me. So I can’t function as the way I would like to be at my age, which is [XXX]. So I cannot go to a job. I do not hold a job. I, I cannot exercise three or four times a week consistently. I cannot cook food every day. I have to eat, you know, frozen meals sometimes. I cannot go do errands every time that I want to go do errands. Um, I cannot be social when I want to be social.”

**Fig. 1:**
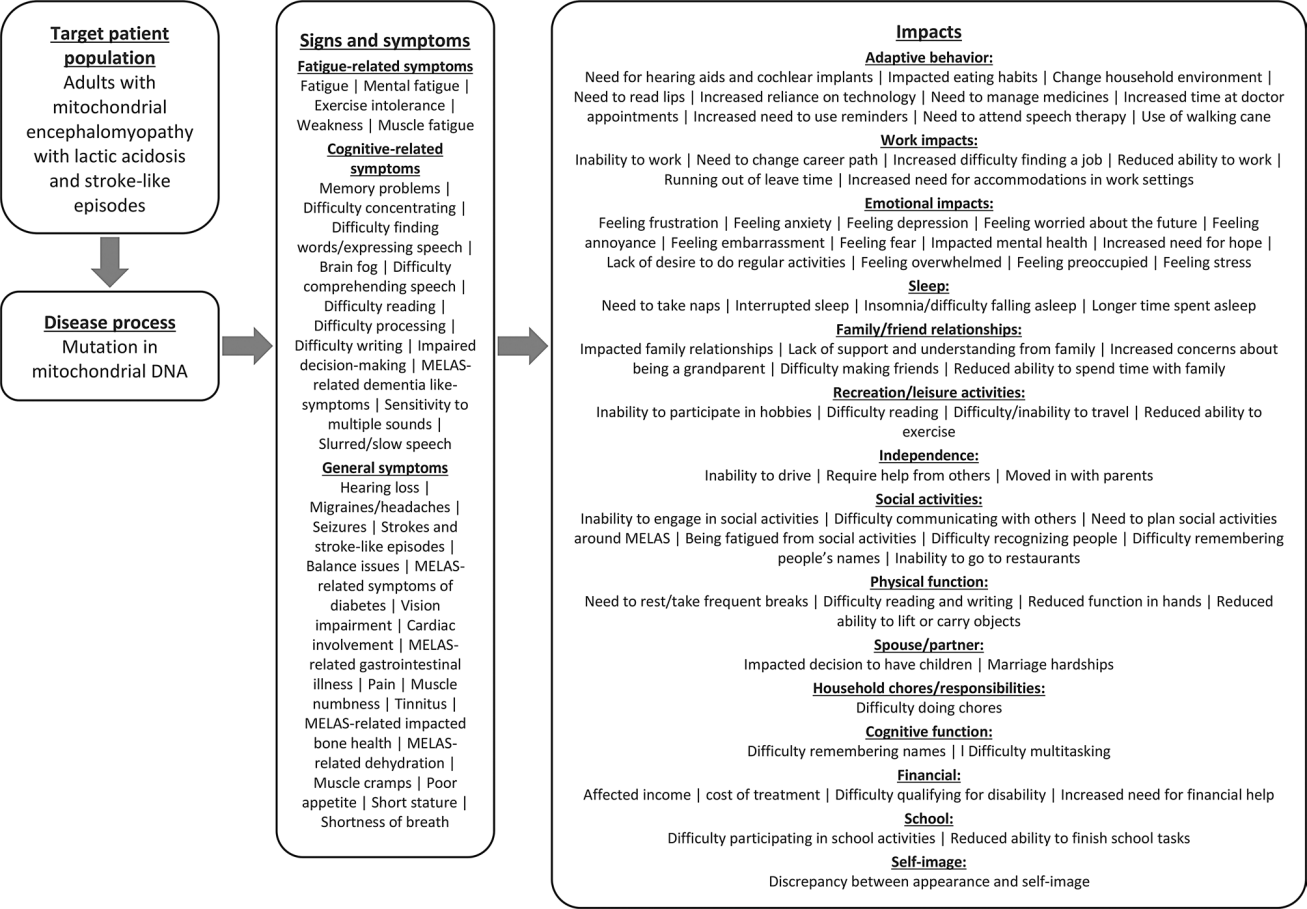
Patient-centric MELAS conceptual model

For concept descriptions and frequencies of all concepts, please see Appendix C. For additional exemplary participant quotes, see Table 4. All concepts reported by participants and clinicians were organized into an adult patient-centric conceptual model which outlines all symptoms, impact domains, and the impact concepts within each impact domain (Fig. 1).

**Table 4:** Exemplary quotes representing the most common impact concepts*

Concept	Exemplary quotes
Adaptive behaviors (*n* = 14)	
Need for hearing aids and cochlear implants (*n* = 10)	**01–02** “Um, I have two hearing aids. Um, and, um, I have, um, good technology on my, my phone where I can keep up with, but it’s very, very hard, um, having MELAS and having hearing aids because it gets really hard when you are trying to have conversations with people and you don’t understand anything that the person is saying, and so you already have comprehension issues and you have hearing issues, so it’s very hard to have conversations with people.” **01–05** “When I was in my mid-twenties…that’s when I started wearing the hearing aids…. Uh, with the cochlear implant, I generally can understand conversation. If there’s let’s say excessive noise, excessive noise would be at restaurants that are crowded. I wouldn’t probably understand, uh, the waiter or waitress.” **01–23** “I ended up getting my first hearing aid about 15 years ago, maybe a little bit less than 15 years ago…. And then I had another hearing test last year and it showed that both my ears are progressively much worse than they were before. So now I have – um, I got my hearing – both hearing aids, a new hearing aid in November of this past year.”
Work impacts (*n* = 14)	
Inability to work (*n* = 8)	**01–01** “Well I feel like if you are fatigued, in general if a person is, is fatigued like too much, that impedes your ability to participate in what you want to do. And that, that’s the case for me. So I can’t function as the way I would like to be at my age…. So I cannot go to a job. I do not hold a job. I, I cannot exercise three or four times a week consistently. I cannot cook food every day.” **01–02** “Um, I’m actually an attorney and, um, a teacher. And I, um, couldn’t do either one, um, and I still can’t do either one. Um, they diagnosed me with MELAS. I went through speech therapy for two years because they diagnosed me with aphasia. Um, and I was having – I’m sorry. Cognitive behaviour – I mean, um, comprehension issues.” **01–16** “Uh, but the stroke, uh, that I had in 2022, uh…I’m not working… I am not, uh, capable at this moment of even, uh, going close to where I was, uh, prior to even 2019.” **01–21** “I can’t work. I can’t travel… And, um, I was there with the company for 22 years. And I loved it. It was fabulous and I enjoyed it. And I was a very senior person, um, and I, you know, I, I miss it so much now ‘cause I, I can’t travel and I can’t work right now.”
Need to change career path (*n* = 4)	**01–15** “I, uh, I’ve always been in positions that are retail that involve a lot of like stocking shelves and moving merchandise and things like that. Um, so with my, with my last job, I, I was managing a, uh, store facility.… And that became too much for me to do. Um, like to have that arm situation with the sleeping and I realized I, I can’t do any jobs that I’ve ever done that I’ve ever been qualified to do that anybody would even consider hiring me for. So I went back to school so that I could do something with computers that involves me sitting at a desk.” **01–16** “I guess this also ends up changing what I end up just thinking about because when I ended up getting, uh, tested for MELAS, uh, my doctor, uh, recommended that I don’t end up, uh, finding a job that could be stressful. Uh, leading up to, uh, my MELAS, uh, I was an attorney.… And, uh, she recommended that I no longer be an attorney because of, uh, the stress that MELAS can, uh, create.…” **01–27** “At first it, uh – I mean, um, when you’re a fire-fighter, it’s just not you alone. It’s the people you work with and things like that. And, um, I mean you’re in the middle of a medical or even a major incident and if you have – like I mean in the middle of it and have a stroke-like incident or anything happens to you, it affects the people majorly. And I wouldn’t want anyone to be put in that kind of a situation, life-threatening kind of situation. So I mean it wasn’t just like a decision for me. It was the decision of like something happening, like causing an incident inside of an incident.… And that was the first decision not being a fire-fighter anymore.”
Emotional function (*n* = 13)	
Feeling frustration (*n* = 4)	**01–05** “Yeah. There is frustration….I used to be able to do something like this. I can no longer do it. Whether it’s walking around the house…. because I don’t have the proper balance to stand on the ladder and do that. So those are frustrations or whatever. Being dependent on others. Um, going to a food buffet and not being able to identify the food it’s like you take what you can get.… So frustration, being dependent on someone else to give me a ride and their time schedule and their ability having to be ready for them and maybe they show up, maybe they’re really late. Uh, having to identify the proper car. Those are all frustrations.” **01–15** “There has definitely been some days where you, you just kind of break down and cry ‘cause it’s frustrating. INTERVIEWER: And, um, what aspects specifically are frustrating? PARTICIPANT: Watching my independence flee.”
Sleep (*n* = 11)	
Need to take naps (*n* = 6)	**01–20** “Um, it varies in terms of what level of fatigue I have. Um, it may be the result of a sleepless night, um, or it could be after I experience something stressful, um, or anxiety. And if I have the ability, um, during the daytime, I take a nap. Um, and when I do, um, I really, um, really fall asleep so that, um, for two hours or so, um, um, I will be in deep sleep. Um, if I, if I take a nap during the day.” **01–27** “Uh, uh, obviously the biggest one for me is like fatigue. Um, like if I get too over like exhausted or anything like that, it really takes it out of me. Uh, my favourite hobby is taking naps…. Um, uh, right now I’m in between jobs but, uh, definitely like, uh, um, when I was working, uh, I would get home from work and, uh, even before dinner, I would have to lie down and take a nap just to get my energy up and running and whatnot.… INTERVIEWER: Okay. Um, so when you talk about taking naps, how often would you say that you take a nap? PARTICIPANT: Probably every day.” **01–28** “Um, I usually take a nap in the afternoon, like pretty much every day, even if I slept well, which I don’t usually sleep very well, which is probably why I’m napping all the time.…”
Family/friend relationships (*n* = 10)	
Impacted family relationships (*n* = 7)	**01–07** “And it’s affected interpersonal relationships with family and friends.… I think my daughter has it, but she won’t test. So she just stops talking for months and years.… Stops talking to me. Stops talking to her friends. She quit her job. It’s kind of frightening what goes on.… It’s basically a loss of, of like stability and family units and …” **01–22** “I pretty much lost my, um, relationship with my family. But, um, because of my disease and – …Probably like to, but, um, can’t do anything so they, um, they look down on me and, um …” **01–25** “Um, there’s definitely like some riffs I feel like that happened and, um, yeah. We recently had another family member pass who also has MELAS, um, and she was, I don’t know, pretty young. I mean my grandmother died when she was, uh, [XXX], something like that. Um, and my great-aunt just died and she was around the same age. So that’s a little freaky, but other than that I think it was just very emotionally taxing on all of us in different ways.”
Recreation/leisure activities (*n* = 10)	
Inability to participate in hobbies (*n* = 5)	**01–01** “Um, I don’t do any physical activities. You know, I can’t do like hiking or, um, longer walking experiences such as I went to the Grand Canyon with my son and I had a difficult time walking through that. Not into the canyon, but, you know, on the path. I had a difficult time with that. I, I, I did it, but it was very difficult and very painful on my muscles. So that kind of stuff, yes. Um, my hearing affects my ability to eat at a restaurant because of the – well the noise. I can’t hear. Um, so physically I can’t do a lot of physical activities. I can manage stuff like a movie if there is open caption on the screen. Um, I can manage if I’m one-on-one with a person in a quiet environment and we’re not doing anything terribly physical.” **01–20** “Um, well, um, because I mentioned that when I was with the bike group, um, apparently I fell and had experienced a seizure…. I, I realized I, I couldn’t remember how to say my home address or say the word key that I needed to say. Um, to the other people who I was riding with…. So, um, so that has meant that I kind of stopped riding my bike….So, um, um, so I would just, yeah, I would just mention that, um, I am reluctant to ride my bike because the experience has given me certain worries of balance. **01–22** “Pretty much everything that I had to just – I can’t do anymore. Yeah. I can’t do a lot of my hobbies.… CAREGIVER: Most of your hobbies. If, if he is to do any hobbies at this point, he needs my assistance with almost everything, right? PARTICIPANT: Yeah. CAREGIVER: With almost everything. If I can support him, he can do short, 5–20 minutes at a time of like going fishing, but I have to drive him and I have to put the stuff together and, and then he stays for like – he’s super fatigued for probably a week after.”
Independence (*n* = 9)	
Inability to drive (*n* = 6)	**01–02** “Um, here in [XXX] when you have seizures, um, you legally cannot drive for at least six months. So, um, I have a, a nice, um, um, convertible and I couldn’t even drive my car.… And sometimes I may be in my car and some – and I – and have – it scares me ‘cause then I may have a headache out of the blue while I’m driving.” **01–06** “I can’t drive. I’ve tried so many times to get my driver’s license and like I’ve gone to classes.… It like literally affects every single part of my life…” **01–28** “Um, I don’t drive anymore because of the, the seizures and the migraines, but mostly the seizures. That I get the myoclonic seizures. So friends come to me or I get rides.”
Require help from others (*n* = 6)	**01–02** “Um, and because I have hearing aids too and because I have comprehension issues, um, when I go to my doctor’s offices, um, that’s hard too. Um, and so sometimes I have to have my husband take – go with me because I need an, um, uh, uh, advocate, you know, who can actually hear what has been said because sometimes I don’t understand it. Um, so it’s hard having two hearing aids too, so…” **01–05** “Being dependent on others. Um, going to a food buffet and not being able to identify the food it’s like you take what you can get.… So frustration, being dependent on someone else to give me a ride and their time schedule and their ability having to be ready for them and maybe they show up, maybe they’re really late.” **01–15** “And the independence, you know, if I’ve got to hold off certain tasks until my husband is around or somebody else is around that can help me, then that’s more time wasted.”
Social activities (*n* = 8)	
Inability to engage in social activities (*n* = 5)	**01–02** “I don’t get with my friends and stuff, um, because, you know, I can’t engage. And, um, you know, they may be laughing and having conversations and all that. And so unfortunately, yeah, I just don’t feel comfortable, um, being, um, in my groups anymore. But just – it’s – which is sad because, you know, before all this, um, yeah, I would be with my friends and, you know – but, yeah. So now it’s just pretty much just me and my husband, um, do things together and stuff. Um, so, yeah, socially I, I – my social life pretty is not good.… Sometimes I do mentally just get, get tired and just, just don’t even want to be bothered by people…” **01–22** “Um, I’m not able to, um, to go to all the activities. Um, um, it’s hard to explain to them because they don’t understand, um, how tired I am and, um, it really wears – um, to wear me out, um, being around people, um, and, um, um, they don’t understand, uh, my disease. Um, I try to, um – my dad understand a little bit that he explains but they don’t understand, um, my condition, um, how it wears me out and I’m so tired.” **01–24** “Um, and I guess it’s slightly off your question, but to add to that I mean just like being in pain – so I’m in some level of pain almost all the time from like either muscle soreness or a muscle like injury or headaches or, um, and that affects like, you know, how much I want to like be social and how I show up at work and all of the, all of the things just a pain in the ass.”
Physical function (*n* = 7)	
Need to rest/take frequent breaks (*n* = 4)	**01–02** “And so, um, I try to walk and stuff, but you just get too tired. Yeah. You’re very fatigued and tired… Um, like if I’m in an, um, airport, I cannot walk down, um, to where I’m supposed to be. I usually just stop and maybe sit for a minute and then keep walking and – or, um, here at the house, we’ll try to like walk down our, um, street to walk back up and that’s about it.” **01–15** “Um, if we know that there’s a fun activity we want to be doing, uh, coming up on a weekend, then we make sure that I’m resting for like the day before, the day after so that I’ve got enough energy to go through that. Nobody ever asks us to do anything spur of the moment.”
Spouse/partner relationship (*n* = 7)	
Impacted decision to have children (*n* = 6)	**01–22** “All my – all the plans of my future, uh, like a career, um, we had to change, um, our plans, um, our plans for my future and, um, yeah. We’d love to have kids, but, um, it’s, um – our plans changed because of my disease.” **01–23** “Um, my decision to have children was a long time ago. I don’t plan to have any children because I don’t want to give MELAS to them. If I already know the effects that could possibly have, I knew a long time ago that I don’t want to watch them suffer.” **01–25** “Um, and then also probably the biggest thing, which happens – it’s less of a thing I think about now, but when I first found out about having MELAS, like, um, just kind of thinking that like I kind of couldn’t have kids because I would pass it down to them, which is a thing that I think about sometimes. That’s probably the biggest long-term effect.… I mean I’m not really at a point in my life where like I think about it all the time because I’m not looking to have kids right now. But I think it is something – it will be something I definitely think about when that time comes for sure.” **01–28** “Um, I had tubal ligation two years ago because of the maternal passing it down. I didn’t want my potential kids to deal with this.”
Household chores/responsibilities (*n* = 6)	
Difficulty doing chores (*n* = 6)	**01–05** “Normal daily activities that should be done, affected them. Something can be sitting on my table that shouldn’t be there and tossed away and maybe it sits there for three weeks because I’m just too tired to deal with, with things. So whatever. It’s like, yeah. I deal with taking care of my pet. That’s a priority because the pet can’t take care of itself. You know, as far as getting her out for walks and whatever or feeding it. To plan meals for myself. Yes. Everything else is optional when it gets done. And it doesn’t get done as quickly or soon as it should because of fatigue.” **01–07** “Yeah. Yeah. Sometimes I have bad days and I’m not, not going to be able to walk to the mailbox.” **01–22** “Yeah. ‘Cause I start it and then, um, it’s either like a day later or, um, sometimes like a week, I’m working so I’m outside and, um, I’ve got to take a break for like a day or, um, yeah, it’s hard to do start to finish something. Mainly like an hour I do something small and it takes like a half hour, an hour later.”

^*^Defined as reported by ≥ 4 participants

## Discussion

This study aimed to develop an understanding of the patient experience of MELAS through the conduct of qualitative interviews with expert clinicians and patients with MELAS.

Clinicians reported that MELAS is a progressive disease, with an array of debilitating symptoms that affect patients’ ability to live a normal life. These findings were largely reiterated by the findings from the patient interviews. On the whole, the breadth, severity, and inter-participant variability of symptoms and impacts reported by both experts and patient themselves indicate that MELAS manifests heterogeneously and affects multiple aspects of a patient’s life including their physical health, their emotional well-being, their daily activities, and their livelihood.

One area where clinician and patient reports diverged was in the differing emphasis on multi-organ involvement and acute symptoms. Clinicians emphasized broader organ system involvement and acute neurological symptoms (e.g., stroke-like episodes) while patients more frequently focused on the chronic symptoms of MELAS that impact their day-to-day functioning. Though there are no therapies approved for the treatment of any aspect of MELAS, current treatment approaches, however limited, are aimed at managing the more acute manifestations of MELAS (e.g., seizures, migraines, and stroke-like episodes [26]), as reflected in the reports of expert clinicians. Unfortunately, among the limited options currently available, none are aimed at alleviating the chronic symptoms that patients with MELAS report as most important to improve, such as physical fatigue.

While patients and clinicians each reported a wide range of MELAS symptoms, the symptoms that were spontaneously and overwhelmingly reported by both were related to fatigue and cognition, including, but not limited to, physical fatigue, mental fatigue, exercise intolerance, memory problems, and difficulty concentrating. In studies of the broader PMD population, fatigue has consistently been the most frequent and the most bothersome symptom reported [11]. Similarly, in this study of patients with MELAS, fatigue-related symptoms were also among the most frequently reported and were identified as most bothersome and most important to improve. However, cognition-related symptoms were reported by patients with MELAS nearly as frequently as fatigue-related symptoms. This finding reflects the unique CNS pathophysiology of MELAS, which makes it distinct from other mitochondrial disease syndromes that do not present with stroke-like episodes. The impact of MELAS on both peripheral systems, including muscle, and the CNS is consistent with the fatigue-related and cognition-related symptoms reported by patients. This constellation of symptoms profoundly impacts the ability of patients with MELAS to participate in important life activities and complete daily tasks.

Mental fatigue, which was reported by 75% of patients in this study, exemplifies the complex intersection of fatigue and cognition experienced by patients with MELAS. Patients attributed cognitive challenges, such as the inability to complete and concentrate on tasks, to “mental fatigue.” However, mental fatigue itself is a multifaceted concept that can embody several cognitive symptoms and neuropsychological concepts simultaneously. Review of patient interview statements suggests that the challenges ascribed to mental fatigue may be more precisely ascribed to deficits in executive function, processing speed, and/or attention, in addition to an overall decrease in mental energy and stamina. The same can be said for memory problems, a similarly multifaceted concept that upon closer review is supported by patient statements that include examples of word-finding difficulties and deficits in episodic memory, attention, and working memory. Not surprisingly, patients use terms to reflect their experience of the condition, rather than clinical terms that specifically identify the neuropsychological processes underlying those experiences. Nevertheless, patients can accurately report both their symptoms and the associated impacts to their life in meaningful terms.

As MELAS is a rare disease within the broader umbrella of rare mitochondrial diseases, research focused on MELAS has been limited, especially research on the patient perspective. While the perspectives of patients with primary mitochondrial diseases have been described [11–14], the findings from this research should inform future research on MELAS as well as on PMDs more generally through the report of the concepts most important to patients with MELAS. Clinical trials should reference these results to guide selection of appropriate instruments for the measurement of concepts that are relevant and important to patients.

Given the frequency and importance of the multifaceted cognitive symptoms and impacts identified in this interview study, in addition to PRO assessments, consideration should be given to the use of performance-based outcome (PerfO) assessments of cognitive function in this population. PerfO assessments may be appropriate given the worsening of cognitive impairment over time [4, 5] and the heterogeneity in symptoms and impacts identified [27]. In addition, expert review of patient statements, incorporating neuropsychological and cognitive science frameworks, may be necessary to guide PerfO measure selection.

### Limitations

There are several limitations to consider for this work. First, to be eligible for this study, participants had to be adults who were able to provide informed consent; able to read, write, and comprehend English; and able to participate in two 45-minute interviews. Additionally, most participants in this study population were well educated, with 75.0% (*n* = 12/16) reporting an undergraduate or post-graduate degree, which suggests later disease onset. Thus, the voices of children and patients with earlier disease onset as well as those with more severe cognitive impairment were not captured. Second, although the eligibility criteria were restricted to adult participants who could complete interviews without the assistance of a caregiver, two participants had caregivers who assisted during their interviews and these patients’ responses were included in the analysis. Another potential limitation is that the sample was not stratified by disease severity or time since disease onset. As participants with younger onset and longer duration may have more advanced MELAS than those with later onset, this may lead to differences in experience not captured in this report. Finally, as with all studies, participants self-selected into this study; therefore, there may be other eligible patients with potentially different signs, symptoms, and HRQoL impacts who chose not to participate. However, we believe data from the expert interviews helped capture the profile of the more complete population of patients with MELAS.

## Conclusion

In summary, the qualitative data from interviews with expert clinicians and patients with MELAS substantiate that MELAS manifests heterogeneously and profoundly affects multiple aspects of a patient’s life. These results also establish that both fatigue and cognitive symptoms are common to the patient experience of MELAS, and that these daily symptoms are considered both most bothersome and most in need of an effective treatment by patients with MELAS. Accordingly, the concepts of fatigue and cognitive impairment should be prioritized for assessment in MELAS clinical trials.

## Electronic Supplementary Material

Below is the link to the electronic supplementary material.

Appendix A: Expert concept description tables

Appendix B: Saturation

Appendix C: Patient concept description tables

## Data Availability

Anonymized data and related documentation underlying the reported results will be shared with qualified investigators whose proposal of data use has been approved by a sponsor review committee.
