# Supplementary material for: Signs, symptoms, and health-related quality of life in MELAS: measuring what’s important from the patient and clinician perspectives
Source: J Patient Rep Outcomes. 2025 Oct 27;9:127. doi: 10.1186/s41687-025-00962-6 (PMC12559487; doi:10.1186/s41687-025-00962-6)
Supplement: Supplementary file 1 — Appendix A: Expert concept description tables [file 41687_2025_962_MOESM1_ESM.docx]

| **Table 1. Expert-reported sign and symptom concept description table** | | | |
| --- | --- | --- | --- |
| Concept | Concept description^*^ | Total frequency of expert report  N=5  n (%)^†^ | Most concerning to clinicians^‡^ |
| Physical fatigue | Experts described physical fatigue as a feeling of tiredness and weakness associated with decreased activity levels; less tolerance for social activities or work/school-related activities; limited ability to leave the house to go shopping or go to school/work; sleeping 11–12 hours per night but still waking up tired or needing to take naps during the day | 5 (100.0%)  *Spontaneous: 5 (100.0%)*  *Probed: 0 (0.0%)* | 3 |
| Seizures | Experts described seizures as a neurological manifestation of MELAS that often co-occurs with strokes or stroke-like episodes; experts described seizures as more common than stroke-like episodes, but not experienced by all patients | 5 (100.0%)  *Spontaneous: 5 (100.0%)*  *Probed: 0 (0.0%)* | 1 |
| Stroke/stroke-like episodes | Experts described strokes/stroke-like episodes as a neurologic manifestation of MELAS that may or may not occur in patients and can be identified based on an abnormal magnetic resonance imaging; experts described this concept as bothersome and concerning to patients, and stated that it can result in vision loss, decreased cognitive capabilities, balance issues, dementia, hearing loss, and/or co-occurring seizures/epilepsy; experts described it as a predictor of worsening neurological symptoms with age | 5 (100.0%)  *Spontaneous: 5 (100.0%)*  *Probed: 0 (0.0%)* | 2 |
| Exercise intolerance | Experts described exercise intolerance as a difficulty walking long distances or climbing stairs; need to rely on mobility aids such as scooters or wheelchairs to complete errands; can be too fatigued for other daily activities after getting dressed, bathing, and eating; associated with physical deconditioning | 5 (100.0%)  *Spontaneous: 5 (100.0%)*  *Probed: 0 (0.0%)* | 2 |
| Memory loss | Experts described memory loss as difficulty remembering in day-to-day life; associated with stroke-like episodes, which can cause damage to memory; described as slowness of thoughts, and similar to dementia; impacts independence and ability to be left alone without a caregiver | 5 (100.0%)  *Spontaneous: 4 (80.0%)*  *Probed: 1 (10.0%)* | 3 |
| Mental fatigue | Experts described mental fatigue as feeling overwhelmed more easily, making errors during day-to-day activities, engagement in conversations or activities tapers off after about fifteen minutes, short attention span; described as similar to and difficult to differentiate from executive dysfunction | 5 (100.0%)  *Spontaneous: 5 (100.0%)*  *Probed: 0 (0.0%)* | 0 |
| Impaired executive function | Experts described impaired executive function as difficulty with prioritizing, contextualizing, organizing, and planning; can result in an inability to put thoughts into actions or words; students may struggle to take a full course load of classes due to difficulty with studying and taking notes; may appear in the middle stage of disease progression | 5 (100.0%)  *Spontaneous: 4 (80.0%)*  *Probed: 1 (10.0%)* | 1 |
| Cardiac involvement | Experts described cardiac involvement as cardiomyopathy, heart failure, or other heart abnormalities (e.g., arrhythmias) that may require heart transplantation; 1/3 of MELAS patients were described as being at-risk for developing cardiomyopathy | 5 (100.0%)  *Spontaneous: 5 (100.0%)*  *Probed: 0 (0.0%)* | 0 |
| MELAS-related diabetes | Experts described MELAS-related symptoms of diabetes as being relatively manageable using oral or injectable medication and often co-described with hearing loss or deafness; experts described this as occurring without obesity and often being maternally inherited | 5 (100.0%)  *Spontaneous: 5 (100.0%)*  *Probed: 0 (0.0%)* | 0 |
| Hearing loss | Experts described hearing loss as either “hearing loss” or “deafness” and often co-described with diabetes; experts described hearing loss as often being maternally inherited; most patients will use hearing aids; some develop sensorineural hearing loss and lose the ability to communicate | 5 (100.0%)  *Spontaneous: 5 (100.0%)*  *Probed: 0 (0.0%)* | 0 |
| Migraine | Experts described migraines as recurrent and severe headaches resulting in nausea, vomiting, visual aura, and brain fog | 5 (100.0%)  *Spontaneous: 5 (100.0%)*  *Probed: 0 (0.0%)* | 0 |
| Impaired visuospatial abilities | Experts described impaired visuospatial abilities as a lack of situational orientation in space and time; difficulty with navigation or following instructions; changes to the field of vision including distortions and interference; associated with damage from stroke-like episodes | 5 (100.0%)  *Spontaneous: 1 (10.0%)*  *Probed: 4 (80.0%)* | 0 |
| Difficulty concentrating | Experts described difficulty concentrating as difficulty paying attention and slowing of processing speeds; reduced ability to think clearly; associated with cognitive fatigue and intellectual disabilities; can manifest as poor work- or school-performance | 4 (80.0%)  *Spontaneous: 4 (80.0%)*  *Probed: 0 (0.0%)* | 0 |
| Gastrointestinal dysmotility | Experts described gastrointestinal dysmotility as slowing of the gut motility that can cause issues with constipation; some patients take fiber supplements to regulate bowel movements | 4 (80.0%)  *Spontaneous: 4 (80.0%)*  *Probed: 0 (0.0%)* | 0 |
| Headaches | Experts described headaches as a persistent core symptom that can co-occur with migraines and is common in MELAS patients; experts described headaches as bothersome to patients and causing them to seek treatment | 4 (80.0%)  *Spontaneous: 4 (80.0%)*  *Probed: 0 (0.0%)* | 0 |
| Impaired decision-making | Experts described impaired decision-making as an impaired ability to plan and use judgement; occurs in later stages of disease progression; can potentially cause harm to patients if they put themselves in dangerous situations due to poor judgement | 4 (80.0%)  *Spontaneous: 2 (40.0%)*  *Probed: 2 (40.0%* | 0 |
| MELAS-related dementia | Experts described MELAS-related dementia as rare but severe memory loss with changes in mood and personality; occurs in severe MELAS patients, after repeated strokes; dementia worsens with each stroke | 4 (80.0%)  *Spontaneous: 4 (80.0%)*  *Probed: 0 (0.0%)* | 1 |
| Vision impairment | Experts described vision loss as an inability to see well and as visual deficits associated with migraine (e.g., visual aura), neuromyopathy, retinal disease, or vision loss associated with damage to the occipital lobe due to strokes or stroke-like episodes | 4 (80.0%)  *Spontaneous: 4 (80.0%)*  *Probed: 0 (0.0%)* | 0 |
| Weakness | Experts described weakness as a component of fatigue and can co-occur with stroke-like episodes or occur as a result of stroke-like episodes; weakness described as similar to muscle weakness, low muscle tone, lack of physical stamina, and muscle fatigue | 4 (80.0%)  *Spontaneous: 4 (80.0%)*  *Probed: 0 (0.0%)* | 0 |
| Difficulty gaining weight | Experts described difficulty gaining weight in MELAS patients as struggling to gain weight despite being underweight | 3 (60.0%)  *Spontaneous: 3 (60.0%)*  *Probed: 0 (0.0%)* | 0 |
| Difficulty processing | Experts described difficulty processing as difficulty with complex intellectual tasks, including challenges with reading comprehension and speech | 3 (60.0%)  *Spontaneous: 2 (40.0%)*  *Probed: 1 (20.0%)* | 0 |
| Low body weight | Experts described low body weight as characteristic underweight and difficulty gaining weight, in which adult men with MELAS typically weigh under 130 pounds, and adult women weigh around 100 pounds; experts described this as co-occurring with short stature | 3 (60.0%)  *Spontaneous: 3 (60.0%)*  *Probed: 0 (0.0%)* | 0 |
| Short stature | Experts described short stature as growth issues that present in late childhood or adolescence; often co-described with low body weight and failure to thrive | 3 (60.0%)  *Spontaneous: 3 (60.0%)*  *Probed: 0 (0.0%)* | 0 |
| Brain fog | Experts described brain fog as occurring with mental fatigue | 2 (40.0%)  *Spontaneous: 2 (40.0%)*  *Probed: 0 (0.0%)* | 0 |
| Difficulty finding words/ expressing speech | Experts described difficulty finding words/expressing speech as difficulty getting words out, or difficulty processing speech, as a result of stroke-like episodes | 2 (40%)  *Spontaneous: 2 (40.0%)*  *Probed: 0 (0.0%)* | 0 |
| Learning-disability-like symptoms | Experts described learning-disability-like symptoms as borderline or low IQ scores; difficulty with reading or math; inability to do complex intellectual tasks; can be diagnosed with attention deficit disorder in childhood | 2 (40.0%)  *Spontaneous: 2 (40.0%)*  *Probed: 0 (0.0%)* | 0 |
| MELAS-related kidney failure | Experts described MELAS-related kidney failure as renal failure or kidney issues, which may require dialysis or kidney transplantation as disease progresses | 2 (40.0%)  *Spontaneous: 2 (40.0%)*  *Probed: 0 (0.0%)* | 1 |
| Autism-like symptoms | Experts described autism-like symptoms as poor eye contact and verbal abilities; impaired communication; developmental delays | 1 (20.0%)  *Spontaneous: 1 (20.0%)*  *Probed: 0 (0.0%)* | 0 |
| Balance issues | Experts described balance issues as ataxia and trouble with coordination | 1 (20.0%)  *Spontaneous: 1 (20.0%)*  *Probed: 0 (0.0%)* | 0 |
| Changes in mood | Experts described changes in mood and/or personality that may accompany cognitive decline | 1 (20.0%)  *Spontaneous: 1 (20.0%)*  *Probed: 0 (0.0%)* | 0 |
| Diarrhea | Experts described diarrhea as occasionally alternating with constipation and co-occurring with irritable bowel syndrome | 1 (20.0%)  *Spontaneous: 1 (20.0%)*  *Probed: 0 (0.0%)* | 0 |
| Difficulty reading | Experts described inability to read due to severe cognitive impairment and/or autism | 1 (20.0%)  *Spontaneous: 1 (20.0%)*  *Probed: 0 (0.0%)* | 0 |
| Emotional fatigue | Described only as another category of fatigue | 1 (20.0%)  *Spontaneous: 1 (20.0%)*  *Probed: 0 (0.0%)* | 0 |
| Endocrinopathy | Experts described endocrinopathy as including thyroid disease or parathyroid hormone disease | 1 (20.0%)  *Spontaneous: 1 (20.0%)*  *Probed: 0 (0.0%)* | 0 |
| Gastroparesis | Experts described gastroparesis as causing the stomach not to empty and leading to early satiety; associated with underweight and malnourishment | 1 (20.0%)  *Spontaneous: 1 (20.0%)*  *Probed: 0 (0.0%)* | 1 |
| Hallucinations | Experts described visual or auditory hallucinations; thinking that people are fake and/or imposters | 1 (20.0%)  *Spontaneous: 1 (20.0%)*  *Probed: 0 (0.0%)* | 0 |
| Hemiparesis | Experts described hemiparesis as weakness on one side of the body due to strokes or stroke-like episodes | 1 (20.0%)  *Spontaneous: 1 (20.0%)*  *Probed: 0 (0.0%)* | 0 |
| Irritable bowel syndrome | Experts described irritable bowel syndrome as alternating diarrhea and constipation | 1 (20.0%)  *Spontaneous: 1 (20.0%)*  *Probed: 0 (0.0%)* | 1 |
| Low muscle tone | Experts described low muscle tone or hypotonia that may result from a stroke or stroke-like episode | 1 (20.0%)  *Spontaneous: 1 (20.0%)*  *Probed: 0 (0.0%)* | 0 |
| Muscle pain | Experts described patients experiencing muscle pain | 1 (20.0%)  *Spontaneous: 1 (20.0%)*  *Probed: 0 (0.0%)* | 0 |
| Myopathy | Experts described myopathy as limiting physical activity | 1 (20.0%)  *Spontaneous: 1 (20.0%)*  *Probed: 0 (0.0%)* | 0 |
| MELAS-related sleep apnea | Experts described MELAS-related sleep apnea as an aspect of fatigue in which sleep difficulties including sleep apnea can worsen fatigue in a patient | 1 (20.0%)  *Spontaneous: 1 (20.0%)*  *Probed: 0 (0.0%)* | 0 |
| Muscle fatigue | Experts described muscle fatigue as issues with muscles causing fatigue | 1 (20.0%)  *Spontaneous: 1 (20.0%)*  *Probed: 0 (0.0%)* | 0 |
| Ptosis/eye movement problems | Experts described ptosis as eye movement problems that can be bothersome to some patients by making them appear different | 1 (20.0%)  *Spontaneous: 1 (20.0%)*  *Probed: 0 (0.0%)* | 0 |

^*^Drawn from descriptions of the concepts provided by experts

^†^Number of experts who reported the concept

^‡^Number of experts who reported the concept as being the most concerning to clinicians; counts are reported without percentages to reflect that some experts did not provide responses to each question; counts are not mutually exclusive

| **Table 2. Expert-reported HRQoL impacts concept description table** | | | |
| --- | --- | --- | --- |
| Concept | Concept description^*^ | Total frequency of expert report  N=5  n (%)^†^ | Most concerning to clinicians^‡^ |
| Emotional functioning | | | |
| Feeling depression | Experts described depression as poor emotional health due to being unable to leave their house or participate in social activities as they previously did | 3 (60%)  *Spontaneous: 3 (60.0%)*  *Probed: 0 (0.0%)* | 1 |
| Feeling overwhelmed | Experts described feeling overwhelmed as an inability to deal with things in life and being overwhelmed by small day-to-day activities; experts reported that this was triggered by fatigue | 2 (40%)  *Spontaneous: 2 (40.0%)*  *Probed: 0 (0.0%)* | 0 |
| Feeling worried about the future | Experts described patients being concerned about the level of morbidity and age of mortality associated with MELAS | 2 (40%)  *Spontaneous: 2 (40.0%)*  *Probed: 0 (0.0%)* | 0 |
| Agoraphobia | Experts described agoraphobia as patients not wanting to leave their homes or their family after frequent and prolonged hospitalizations | 1 (20%)  *Spontaneous: 1 (20.0%)*  *Probed: 0 (0.0%)* | 0 |
| MELAS-related bipolar disorder | Experts reported patients experiencing bipolar disorder due to MELAS | 1 (20%)  *Spontaneous: 1 (20.0%)*  *Probed: 0 (0.0%)* | 0 |
| Feeling anxiety | Experts reported anxiety as co-occurring with post-traumatic stress disorder (PTSD) from frequent and prolonged hospitalizations; experts reported anxiety as common in young adult patients | 1 (20%)  *Spontaneous: 1 (20.0%)*  *Probed: 0 (0.0%)* | 0 |
| Feeling frustration | Experts described patients as being frustrated about their worsening cognitive impairment | 1 (20%)  *Spontaneous: 1 (20.0%)*  *Probed: 0 (0.0%)* | 0 |
| Feeling helpless | Experts described helplessness as low quality of life associated with being unable to work, contribute to their family, participate in social activities, or maintain a job/education | 1 (20%)  *Spontaneous: 1 (20.0%)*  *Probed: 0 (0.0%)* | 0 |
| Post-traumatic stress | Experts described post-traumatic stress as occurring within the first year after diagnosis as a result of the effects of prolonged hospitalization, including being in intensive care for multiple weeks, having to be connected to IVs, getting in MRI scanners, and becoming physically deconditioned; experts reported that this is common in teenagers and young adults, and can hinder their interest in participating in research | 1 (20%)  *Spontaneous: 1 (20.0%)*  *Probed: 0 (0.0%)* | 0 |
| Independence | | | |
| Require help from others | Experts described MELAS patients that are in the end-stages of their disease as relying on support from caregivers for activities of daily living including eating, bathing, and using the bathroom; caregiver support was described as increasing as patients’ physical health declines | 5 (100%)  *Spontaneous: 2 (40.0%)*  *Probed: 3 (60.0%)* | 0 |
| Impacted ability to be independent | Experts described patients not being able to be independent due to life-limiting factors of MELAS, for example, not being able to drive and not being able to live independently due to seizures, strokes, or progressive cognitive symptoms. One expert reported that this impact was triggered by cognitive impairment | 1 (20%)  *Spontaneous: 1 (20.0%)*  *Probed: 0 (0.0%)* | 0 |
| Inability to drive | Experts described an inability to drive due to seizures, which impacts the independence of patients by making them reliant on family and unable to live alone | 1 (20%)  *Spontaneous: 1 (20.0%)*  *Probed: 0 (0.0%)* | 0 |
| Work/school | | | |
| Decreased performance at work/school | Experts described patients’ decreased performance at school or in their job due to frequent absences and being unable to perform tasks that they were once able to, such as working with numbers or managing schedules; this impact was reported to be triggered by fatigue and cognitive impairment | 4 (80%)  *Spontaneous: 3 (60.0%)*  *Probed: 1 (20.0%)* | 1 |
| Impacted work/ school activities | Experts described patients needing to modify their work and/or school schedules due to MELAS limitations, for example, only going to classes in the morning | 3 (40%)  *Spontaneous: 3 (60.0%)*  *Probed: 0 (0.0%)* | 0 |
| Inability to go to work/school | Experts described patients needing to quit their jobs and being unable to go to school due to needing to stay home, sometimes due to missing a substantial amount of school due to migraines, vomiting, or other MELAS symptoms. One expert reported that this impact was triggered by fatigue | 2 (40%)  *Spontaneous: 2 (40.0%)*  *Probed: 0 (0.0%)* | 1 |
| Household chores/responsibilities | | | |
| Inability to accomplish daily activities | Experts described patients being unable to accomplish the things that they want to do, including daily life activities; one expert reported that this was triggered by fatigue | 3 (60%)  *Spontaneous: 3 (60.0%)*  *Probed: 0 (0.0)* | 0 |
| Inability to shop | Experts described patients being unable to grocery shop due to extreme fatigue and needing increased support, such as a scooter; one expert reported that this impact was triggered by fatigue | 2 (40%)  *Spontaneous: 2 (40.0%)*  *Probed: 0 (0.0%)* | 0 |
| Sleep | | | |
| Impacted sleep | Experts described patients as needing to sleep many hours a day and/or being unable to sleep, as well as needing a disproportionate amount of sleep; experts reported patients needing 11–12 hours of sleep each night, or needing to take 2- to 4-hour naps during the day; one expert reported that this impact was triggered by cognitive impairment, and two experts reported that this impact was triggered by fatigue. | 4 (80%)  *Spontaneous: 4 (80.0%)*  *Probed: 0 (0.0%)* | 0 |
| Adaptive behaviors | | | |
| Need for hearing aids and cochlear implants | Experts described patients as needing hearing aids or cochlear implants to manage their hearing loss | 2 (40%)  *Spontaneous: 2 (40.0%)*  *Probed: 0 (0.0%)* | 0 |
| Needing a disability placard | Experts described patients as needing a disability placard so that they can park close to their destination. One expert reported that this impact was triggered by fatigue | 1 (20%)  *Spontaneous: 1 (20.0%)*  *Probed: 0 (0.0%)* | 0 |
| Needing a feeding tube | Experts described patients needing a feeding tube due to difficulty swallowing, gastroparesis, constipation, irritable bowel syndrome, and malnourishment due to trouble eating | 1 (20%)  *Spontaneous: 1 (20.0%)*  *Probed: 0 (0.0%)* | 0 |
| Needing to modify work/‌school routine | Experts described patients as modifying their school/work routines to better suit their needs; for example, only taking classes in the morning, taking breaks, and living close to campus | 1 (20%)  *Spontaneous: 1 (20.0%)*  *Probed: 0 (0.0%)* | 0 |
| Needing to use a wheelchair or scooter | Experts described patients as needing mobility aids to manage their fatigue when performing daily activities | 1 (20%)  *Spontaneous: 1 (20.0%)*  *Probed: 0 (0.0%)* | 0 |
| Social | | | |
| Inability to engage in social activities | Experts described patients as being unable to socialize and perform activities with others; one expert reported that this impact was triggered by fatigue | 2 (40%)  *Spontaneous: 2 (40.0%)*  *Probed: 0 (0.0%)* | 0 |
| Increased need for social support | Experts described people with less intrinsic resiliency as needing increased social support to manage the circumstances of their disease | 1 (20%)  *Spontaneous: 1 (20.0%)*  *Probed: 0 (0.0%)* | 0 |
| Activities of daily living | | | |
| Impacted personal hygiene | Experts described patients as being unable to perform hygiene activities without caregiver support, including bathing and being clothed | 2 (40%)  *Spontaneous: 1 (20.0%)*  *Probed: 1 (20.0%)* | 0 |
| Impacted dressing | Experts described patients as being unable to dress themselves and needing assistance to dress appropriately | 2 (40%)  *Spontaneous: 2 (40.0%)*  *Probed: 0 (0.0%)* | 0 |
| Impacted eating | Experts described patients as needing assistance to eat | 1 (20%)  *Spontaneous: 0 (0.0%)*  *Probed: 1 (20.0%)* | 0 |
| Physical functioning | | | |
| Impacted mobility | Experts described patients as having impaired mobility and needing assistive devices for support | 2 (40%)  *Spontaneous: 1 (20.0%)*  *Probed: 1 (20.0%)* | 1 |
| Relationships | | | |
| Impacted relationship with spouse | Experts described patients as experiencing conflict or separations in marriages due to the burden of MELAS as a chronic, progressive disorder | 2 (40%)  *Spontaneous: 2 (40.0%)*  *Probed: 0 (0.0%)* | 0 |
| Inability to contribute to relationships | Experts described patients as having a low quality of life because they are not able to work or contribute to their families | 1 (20%)  *Spontaneous: 1 (20.0%)*  *Probed: 0 (0.0%)* | 1 |
| Caregiver responsibilities | | | |
| Inability to care for children | Experts described patients as not being able to care for their children due to fatigue. One expert reported that this impact was triggered by fatigue. | 1 (20%)  *Spontaneous: 1 (20.0%)*  *Probed: 0 (0.0%)* | 0 |
| Cognitive function | | | |
| Cognitive decline | Experts described patients as being unable to do math and having poor visual memory due to an increased number of stroke-like episodes | 1 (20%)  *Spontaneous: 1 (20.0%)*  *Probed: 0 (0.0%)* | 0 |
| Inability to remember to eat | Experts described patients as needing assistance to remember to eat and needing to be encouraged to eat | 1 (20%)  *Spontaneous: 1 (20.0%)*  *Probed: 0 (0.0%)* | 0 |
| Financial | | | |
| Inability to remember to pay bills | Experts descried patients as needing assistance remembering to pay their bills | 1 (20%)  *Spontaneous: 1 (20.0%)*  *Probed: 0 (0.0%)* | 0 |
| Recreation/leisure activities | | | |
| Inability to participate in hobbies | Experts described patients as being inhibited from participating in the activities that they like to do; one expert reported that this impact was triggered by fatigue | 1 (20%)  *Spontaneous: 1 (20.0%)*  *Probed: 0 (0.0%)* | 0 |

^*^Drawn from descriptions of the concepts provided by experts

^†^Number of experts who reported the concept

^‡^Number of experts who reported the concept as being most concerning to clinicians; counts are reported without percentages to reflect that some experts did not provide responses to each question; counts are not mutually exclusive
