# Supplementary material for: Signs, symptoms, and health-related quality of life in MELAS: measuring what’s important from the patient and clinician perspectives
Source: J Patient Rep Outcomes. 2025 Oct 27;9:127. doi: 10.1186/s41687-025-00962-6 (PMC12559487; doi:10.1186/s41687-025-00962-6)
Supplement: Supplementary file 2 — Appendix B: Saturation [file 41687_2025_962_MOESM2_ESM.docx]

Figure 1 Spontaneously reported symptom saturation graph^*^

^*^Please note that concepts are noted cumulatively across participants

Figure 2 Spontaneously reported impact saturation graph*

^*^Please note that concepts are noted cumulatively across participants

| **Table 1. Symptom and impact domain saturation grid** | | | | |
| --- | --- | --- | --- | --- |
| **Root concept/Domain** | **Total sample (N=16)** | | | |
|  | **Transcript group 1 (n=4)** | **Transcript group 2 (n=4)** | **Transcript group 3 (n=4)** | **Transcript group 4 (n=4)** |
| Symptoms | | | | |
| Fatigue | X |  |  |  |
| Exercise intolerance | X |  |  |  |
| Mental fatigue | X |  |  |  |
| Weakness | X |  |  |  |
| Muscle fatigue | X |  |  |  |
| Memory problems | X |  |  |  |
| Difficulty concentrating |  | X |  |  |
| Difficulty finding words/expressing speech |  | X |  |  |
| Brain fog | X |  |  |  |
| Difficulty comprehending speech | X |  |  |  |
| Difficulty reading |  | X |  |  |
| Difficulty processing |  | X |  |  |
| Difficulty writing | X |  |  |  |
| MELAS-related dementia-like symptoms | X |  |  |  |
| Slurred/ slowed speech | X |  |  |  |
| Hearing loss | X |  |  |  |
| Migraines/headaches | X |  |  |  |
| Seizures | X |  |  |  |
| Strokes and stroke-like episodes |  | X |  |  |
| Balance issues | X |  |  |  |
| MELAS-related symptoms of diabetes | X |  |  |  |
| Vision impairment | X |  |  |  |
| Cardiac involvement | X |  |  |  |
| MELAS-related gastrointestinal issues |  | X |  |  |
| Pain | X |  |  |  |
| Muscle numbness |  | X |  |  |
| Tinnitus |  |  | X |  |
| Muscle cramps | X |  |  |  |
| Shortness of breath | X |  |  |  |
| Impact domains | | | | |
| Adaptive behaviors | X |  |  |  |
| Work impacts | X |  |  |  |
| Emotional function | X |  |  |  |
| Family/friend relationships | X |  |  |  |
| Sleep | X |  |  |  |
| Recreation/leisure activities | X |  |  |  |
| Independence | X |  |  |  |
| Social activities | X |  |  |  |
| Physical function | X |  |  |  |
| Cognitive function | X |  |  |  |
| Household chores/responsibilities | X |  |  |  |
| Financial |  | X |  |  |
| School impacts | X |  |  |  |
| Self-image | X |  |  |  |
| Spouse/partner |  | X |  |  |
