# Supplementary material for: Signs, symptoms, and health-related quality of life in MELAS: measuring what’s important from the patient and clinician perspectives
Source: J Patient Rep Outcomes. 2025 Oct 27;9:127. doi: 10.1186/s41687-025-00962-6 (PMC12559487; doi:10.1186/s41687-025-00962-6)
Supplement: Supplementary file 3 — Appendix C: Patient concept description tables [file 41687_2025_962_MOESM3_ESM.docx]

| Table 1. Patient-reported sign and symptom concept description table | | | | |
| --- | --- | --- | --- | --- |
| MELAS-related sign/symptom^*^ | Concept description^†^ | Frequency of participant reports (N=16)^‡^  n (%) | Reported as most bothersome  (N=16)^§^  n | Reported as most important symptom to improve  (N=16)^**^  n |
| Physical fatigue | Participants described being lethargic, tired, and lacking energy to complete tasks (e.g., household chores, exercise, social activities) and/or needing to nap and take breaks | 15 (93.8%) | 6 | 9 |
| Hearing loss | Participants described needing hearing aids or cochlear implants due to being hard of hearing or not being able to hear at all | 13 (81.3%) | 2 | 1 |
| Mental fatigue | Participants described mental fatigue as needing to conserve energy for mental tasks, feeling depleted or tired, and not being able to complete tasks, inclusive of speaking to others and concentrating, or focus on them | 12 (75.0%) | 0 | 0 |
| Exercise intolerance | Participants described a decrease in exercise performance, not having the energy to exercise or keep up with their peers, reduced stamina, needing an increased rest following exercise, and not being able to exercise due to fatigue; participants described exercise as walking, jogging, running, lifting, rock climbing, biking, and cleaning | 11 (68.8%) | 0 | 1 |
| Memory problems | Participants described memory problems as short-term or permanent memory loss wherein they cannot remember names, words, schedules, appointments, etc., and experience overall poorer memory | 11 (68.8%) | 0 | 1 |
| Weakness | Participants described decreased performance, myopathy, and not being able to perform routine tasks without soreness, as well as feeling tired or slow in various areas of their body, such as their hands or feet, from regular or strenuous activity | 10 (62.5%) | 1 | 2 |
| Difficulty concentrating | Participants described having difficulty completing only one task, needing to stop and think about tasks performed, easily forgetting completed tasks, and taking longer to or being unable to keep up with group conversations | 9 (56.3%) | 0 | 0 |
| Migraines/ headaches | Participants described needing to take medication to deal with migraines, as the migraines can vary in severity anywhere between a dull pain and the feeling that one’s brain is burning or that there is a jackhammer being taken to one’s head. Participants reported that the migraines can cause nausea and feel like a stabbing or pressure sensation that may occur along with auras. Participants reported that the migraines may be related to seizures and that they need to sleep to deal with the severity of the migraines. For their experience of headaches, participants described headaches occurring around the head and neck area. If participants experienced both headaches and migraines, they noted that migraines were more severe than headaches. | 9 (56.3%) | 5 | 2 |
| Muscle fatigue | Participants describe muscle fatigue and inability to move and/or lift weights, and muscles not performing as expected | 9 (56.3%) | 0 | 0 |
| Difficulty finding words/ expressing speech | Participants described having difficulty finding the words to convey and express their thoughts; participants described impaired ability to have conversations and resulting feelings of frustration | 7 (43.8%) | 2 | 2 |
| Seizures | Participants described seizures as making their speech slow and slurred, immobilizing them, losing cognition, and being mild to massively severe. Participants described needing to be hospitalized due to seizures, and that the seizures may be caused by a variety of reasons (e.g., lack of sleep, missing medicines, multiple sounds). | 7 (43.8%) | 0 | 1 |
| Strokes and stroke-like episodes | Participants describe stroke-like episodes and strokes as having leg weakness, brain blurriness, tunnel vision, impaired communication, and not being able to walk or speak. Participants described needing to be hospitalized due to stroke-like episodes. | 7 (43.8%) | 1 | 1 |
| Balance issues | Participants described balance issues as leaning or tilting to the side, wobbling when standing, and losing one’s balance without the support of a cane, bed, or chair | 6 (37.5%) | 0 | 0 |
| MELAS-related symptoms of diabetes | Participants described having symptoms of diabetes and insulin-resistance due to MELAS and needing to take insulin and eat a restricted diet | 6 (37.5%) | 1 | 0 |
| Vision impairment | Participants described having a loss of vision in their central vision, near- and farsightedness, double vision, progressive vision loss, and differences in sight ability for each eye. Participants described not being able to see other peoples’ physical features and needing glasses/other vision aids. | 5 (31.3%) | 1 | 0 |
| Cardiac involvement | Participants described experiencing tachycardia, atrial fibrillation, and high heart rate as a result of MELAS | 3 (18.8%) | 0 | 0 |
| MELAS-related gastrointestinal issues | Participants described experiencing constipation, slow bowel motility, and overall digestive issues | 3 (18.8%) | 0 | 0 |
| Pain | Participants described pain and burning as occurring in the muscles and abdomen that may or may not occur following activity, which does not resolve based on massage or stretching, or due to the need to have a bowel movement | 3 (18.8%) | 0 | 0 |
| Brain fog | Participants described brain fog as fuzzy thinking, difficulty concentrating, and having difficulty expressing themselves, as well as not being able to wake up and falling back asleep constantly, as well as spacing out and forgetting things | 2 (12.5%) | 1 | 1 |
| Difficulty comprehending speech | Participants described cognitive-related issues with understanding, processing, and explaining speech when speaking or writing | 2 (12.5%) | 2 | 1 |
| Difficulty processing | Participants described difficulty processing as having difficulty communicating when participating in conversation | 2 (12.5%) | 0 | 0 |
| Difficulty reading | Participants described a significant decrease in reading level that occurred after a stroke or experiencing notable mental fatigue when reading | 2 (12.5%) | 0 | 0 |
| Muscle numbness | Participants described muscle numbness as a tingling feeling or a numb feeling | 2 (12.5%) | 0 | 0 |
| Tinnitus | Participants described experiencing ringing in their ears or a constant “eee” sound in their hearing | 2 (12.5%) | 0 | 0 |
| Difficulty writing | Participant described their writing as appearing like symbols | 1 (6.3%) | 0 | 0 |
| Impaired decision making | Participant described having difficulty making executive decisions when options are presented | 1 (6.3%) | 0 | 0 |
| MELAS-related dementia-like symptoms | Participant describes experiencing dementia-like symptoms (e.g., getting lost, poor memory) | 1 (6.3%) | 0 | 0 |
| Sensitivity to multiple sounds | Participant described being unable to concentrate on and/or understand one sound if there are multiple sounds occurring at the same time | 1 (6.3%) | 0 | 0 |
| Slurred/ slow speech | Participant described slurred and slow speech following and/or during seizures and strokes | 1 (6.3%) | 0 | 0 |
| MELAS-related impacted bone health^††^ | Participant described decreased bone density in their hips due to MELAS | 1 (6.3%) | 0 | 0 |
| MELAS-related dehydration | Participant’s caregiver described the participant being perpetually dehydrated and needing intravenous fluids weekly | 1 (6.3%) | 0 | 0 |
| Muscle cramps | Participant described experiencing severe muscle cramps in various areas of their body | 1 (6.3%) | 0 | 0 |
| Poor appetite | Participant described that nothing tastes good and that they have a decreased appetite | 1 (6.3%) | 0 | 0 |
| Short stature^††^ | Participant did not provide a further description of short stature | 1 (6.3%) | 0 | 0 |
| Shortness of breath | Participant described experiencing shortness of breath when standing up, along with a high heart rate | 1 (6.3%) | 0 | 0 |

^*^Concept reported by study participant

^†^Description of concept based on study participant report

^‡^Frequency is presented as the total number and percentage of study participants who reported each concept at least once

^§^Most bothersome symptom as reported by the study participant, percentages not provided as participants were permitted to report on more than one symptom as most bothersome

^**^Most important symptom to improve as reported by the study participant, percentages not provided as participants were permitted to report on more than one symptom as most bothersome

^††^Classified as a sign, not symptom

| Table 2. Patient-reported HRQoL impact concept description table | | |
| --- | --- | --- |
| Concept | Concept description^*^ | Frequency of participant reports^†^  n (%) |
| Adaptive behavior (n=14/16, 87.5%) | | |
| Need for hearing aids and cochlear implants | Participants described needing devices to help with their hearing such as hearing aids and cochlear implants due to their hearing loss, noting that without they had difficulty or were unable to hear other people speaking, especially in louder settings. One participant also noted needing hearing devices as a result of tinnitus. | 10 (62.5%) |
| Impacted eating habits | Participants described limitations to their diet as a result of MELAS. One participant described needing liquid meals, or occasionally being too tired to chew. One participant described limiting carbohydrates, and one participant described not being able to eat specific foods or any alcohol. | 3 (18.8%) |
| Change household environment | Participants described needing a household environment without stairs, due to lack of stamina. A caregiver reported the participant needing an individual bed and room. | 2 (12.5%) |
| Need to read lips | Participants described depending on reading lips to help with understanding, even with wearing hearing aids in both ears; participants mentioned difficulties understanding others if people’s faces are not visible or covered a mask | 2 (12.5%) |
| Increased reliance on technology | Participants described the use of technology in assisting them. One participant described using Google to help when they were forgetting words. One participant described using text-to-speech and highlighting apps and tools to help accommodate vision loss. | 2 (12.5%) |
| Need to manage medicines | Participants described needing to manage their medication routine, including having their medications on hand at all times being unable to rely on others for assistance, despite the burden of the routine | 2 (12.5%) |
| Increased time at doctor appointments | Participant described having to spend large amounts of time at doctors’ offices, and having multiple doctor’s appointments in a week | 1 (6.3%) |
| Increased need to use reminders | Participant reported having reminders, without which they could not remember things | 1 (6.3%) |
| Need to attend speech therapy | Participant described going to speech therapy for two years after their MELAS diagnosis as a result of their aphasia and difficulty understanding | 1 (6.3%) |
| Use of walking cane | Participant reported sometimes walking with a cane to help them in case they lose their balance | 1 (6.3%) |
| Work impacts (n=14/16, 87.5%) | | |
| Inability to work | Participants described being unable to work or having to quit their jobs as a result of the symptoms of their MELAS, including vision and hearing loss, fatigue, muscle weakness, migraines, and strokes/seizures. One participant attributed being unable to work to fatigue. One participant (and their caregiver) attributed being unable work to cognitive impairment. One participant attributed being unable to work to the overall condition. | 8 (50.0%) |
| Need to change career path | Participants described having to switch careers as a result of physical weakness, medical concerns that stress from their current careers may worsen MELAS, or that a stroke-like incident at work could endanger themselves or others at work. One participant attributed the impact to their career path to the overall condition. | 4 (25.0%) |
| Increased difficulty finding a job | Participants described having difficulty finding positions that would accommodate their condition. One participant also described difficulty in the job search process, attributing this to the overall condition. | 2 (12.5%) |
| Reduced ability to work | Participants described symptoms such as weakness, muscle soreness, headaches, and pain reducing their ability to do work tasks and changing how they work | 2 (12.5%) |
| Running out of leave time | Participants described employment-related issues such as having to call out without notice repeatedly and running out of leave, due to fatigue, migraines, seizures and stroke-like episodes, and dizziness | 2 (12.5%) |
| Increased need for accommodations in work settings | Participant described not being sure how to do work-related tasks without accommodations, such as having difficulty writing a resume, interviewing, applying for a job, and sitting at a desk, as a result of fatigue | 1 (6.3%) |
| Emotional function (n=13/16, 81.3%) | | |
| Feeling frustration | Participants described being frustrated by being dependent on others, being unable to do activities they used to do, and lack of understanding from others; one participant described crying as a result of frustration. One participant attributed their frustration to fatigue. | 4 (25.0%) |
| Feeling anxiety | Two participants described having anxiety as a result of MELAS symptoms; one participant specifically reported feeling anxiety over when and where a seizure might happen. This participant attributed their impacts to both fatigue and cognitive impairment. One participant’s caregiver reported the participant experienced anxiety. | 3 (18.8%) |
| Feeling depression | Participants described a feeling of hopelessness and crying over the state of their health relative to their peers, being unable to do things they wish to do, and the symptoms of MELAS | 3 (18.8%) |
| Feeling worried about the future | Participants described the emotional impact and burden of knowing their life expectancy was impacted by their condition. One participant also described having underlying concerns about whether their conditions was progressing and whether to plan for the future. | 2 (12.5%) |
| Feeling annoyance | Participant described annoyance at having sudden and unexpected headaches | 1 (6.3%) |
| Feeling embarrassment | Participant reported experiencing embarrassment because of the disruption on social interactions, such as not being able to remember names | 1 (6.3%) |
| Feeling fear | Participant reported being afraid due to the life-threatening nature of the condition, and seeing that reflected in tests, as well as symptoms such as stroke | 1 (6.3%) |
| Impacted mental health | Participant reported having a negative impact on their mental health as a result of MELAS, but did not provide a description | 1 (6.3%) |
| Increased need for hope | Participant described being always hopeful that someday research or a treatment will be available, since there are none at the moment | 1 (6.3%) |
| Lack of desire to do regular activities | Participant described not having the energy or desire to do activities they used to do, including tasks such as going outside or feeding animals, noting the lack of desire worsened both seasonally and over time | 1 (6.3%) |
| Feeling overwhelmed | Participant described being overwhelmed when they received the MELAS diagnosis, as well as the information that it affected others in their family and any kids they may have | 1 (6.3%) |
| Feeling preoccupied | Participant described thinking about their condition on a daily basis, and how it has affected their daily habits such as speech and diet | 1 (6.3%) |
| Feeling stress | Participant reported that MELAS itself caused stress, but did not provide a description as to what the stress was like | 1 (6.3%) |
| Sleep (n=11/16, 68.8%) | | |
| Need to take naps | Participants described needing naps regularly due to being tired; some participants described needing naps occasionally, and other reported needing naps daily. A caregiver reported the participant needed naps daily. Two participants and one caregiver attributed the need to take naps to fatigue, and one participant attributed needing to take naps to the overall condition. | 6 (37.5%) |
| Interrupted sleep | Participants described not sleeping through the night due to blood sugar fluctuations or pain | 3 (18.8%) |
| Insomnia/difficulty falling asleep | Participants described having difficulty falling asleep or having insomnia as taking a while, even hours to fall asleep, resulting in not getting to sleep until the early morning. One participant also described not feeling rested after having slept. | 3 (18.8%) |
| Longer time spent asleep | Participants described needing to sleep between nine and fourteen hours a night. | 3 (18.8%) |
| Family/friend relationships (n=10/16, 62.5%) | | |
| Impacted family relationships | Participants described increased pain, rifts, and distance with their families as a result of MELAS; one participant noted they lost their relationship with their family as a result of their MELAS, and one participant reported that MELAS had different impacts on multiple people in their family, leading to increased emotional burden. | 7 (43.8%) |
| Lack of support and understanding from family | Participants described the difficulty explaining or talking about MELAS to family, and being exhausting by a lack of understanding of their condition from others, as most people were unaware of MELAS. One participant also reported feeling frustrated by this lack of support and understanding. | 3 (18.8%) |
| Increased concerns about being a grandparent | Participant described concern about their ability to be a grandparent and guardian for children if they are too tired | 1 (6.3%) |
| Difficulty making friends | Participant reported difficulty making friends as a result of MELAS, but did not describe how or why | 1 (6.3%) |
| Reduced ability to spend time with family | Participant described not spending time with friends and attending social activities due to fear about getting sick in relation to their condition | 1(6.3%) |
| Recreation/leisure activities (n=10/16, 62.5%) | | |
| Inability to participate in hobbies | Participants described being unable to participate in hobbies such as hiking, biking, snowboarding, mountain climbing, running marathons, and golfing. A caregiver noted that they provided assistance with hobbies such as fishing, but it still resulted in fatigue. Both participants and the caregiver attributed the inability to participate in hobbies to fatigue. In addition, one participant also attribution their inability to participate in hobbies to cognitive impairment. | 5 (31.3%) |
| Difficulty reading | Participants (or their caregivers) described being unable to finish books, feeling drained due to having to wear their glasses to read, and having double vision that made it challenging to track words; one participant also described forgetting what they were reading as they were reading it. Two participants attributed their difficulty reading to fatigue, and two participants attributed their difficulty reading to their cognitive impairment. | 3 (18.8%) |
| Difficulty with/ inability to travel | Participants described having limitations on their ability to travel (including both flight and road trips) due to their medications, and the need to plan for medical needs such as doctor’s appointments | 3 (18.8%) |
| Reduced ability to exercise | Participants described having reduced energy to exercise, even though it was a hobby they enjoyed or are still active in, with reduced capacity. One participant attributed their reduced ability to exercise to fatigue. | 3 (18.8%) |
| Independence (n=9/16, 56.3%) | | |
| Inability to drive | Participants, as well as one caregiver, described being unable to drive as a result of seizures, migraines, and vision loss | 6 (37.5%) |
| Require help from others | Participants described needing help from others such as caregivers, spouses, or parents for help with hearing medical information, seeing and acquiring food, remembering things, chores, driving, and taking pills. One participant attributed requiring help from others to fatigue, and one participant described needing help as a result of memory loss. | 6 (37.5%) |
| Moved in with parents | Participant described having to move back in with their parents as a result of multiple hospital stays. | 1 (6.3%) |
| Social activities (n=8/16, 50.0%) | | |
| Inability to engage in social activities | Participants described being unable to participate in social activities or being with other people due to fatigue, pains such as muscle soreness or headaches, difficulty hearing and speaking, and feeling unwell. Three participants attributed inability to engage in social activities to fatigue. | 5 (31.3%) |
| Difficulty communicating with others | Participants described difficulty communicating and engaging in conversation with others, including family, in work settings, and in social settings, due to hearing loss, comprehension issues, and difficulty processing audio. One participant attributed difficulty communicating with others to cognitive impairment. | 2 (12.5%) |
| Need to plan social activities around MELAS | Participants described needing to be aware of both a physical venue’s accommodations, ability to bring and take medications, planning around a medication schedule, and having to specifically create easily modifiable social plans to accommodate their condition and resulting pain | 2 (12.5%) |
| Being fatigued from social activities | Participant described being worn out from social activities and the process of traveling to and from activities, resulting in them not wanting to go as a result. The participant noted they only had the stamina to last two to three hours at a time. Participant attributed this fatigue from social activities to cognitive impairment. | 1 (6.3%) |
| Difficulty recognizing people | Participant described having difficulty in social and professional settings due to vision loss | 1 (6.3%) |
| Difficulty remembering people’s names | Participant described being unable to remember people’s names even after asking multiple times, resulting in embarrassment and difficulty engaging in social interaction | 1 (6.3%) |
| Inability to go to restaurants | Participant described being unable to eat at restaurants as a result of being unable to hear | 1 (6.3%) |
| Physical function (n=7/16, 43.8%) | | |
| Need to rest/ take frequent breaks | Participants described needing to take frequent breaks when engaging in physical activity, including having to lower the intensity of the exercise or having to sit before continuing. One participant described needing to rest prior to any activities, and having reduced spontaneity in order to ensure they have enough energy for the activities. Three participants attributed the need to rest/take frequent breaks to fatigue. | 4 (25.0%) |
| Difficulty reading and writing | One participant described being unable to write as a result of having slow fingers, resulting in needing to type everything. One participant noted they struggled to read, and their caregiver reported that the participant was unable to write, as a result of visual issues and neuropathy. | 2 (12.5%) |
| Reduced function in hands | Participant described being unable to use their right hand for over a month, and requiring time and therapy to train their hand to speed and mobility to a level similar to prior to their stroke | 1 (6.3%) |
| Reduced ability to lift or carry objects | Participant described difficulty lifting or carrying items of even light to moderate weight, such as groceries or packed boxes. Participant attributed their reduced ability to carry or lift objects to fatigue. | 1 (6.3%) |
| Spouse/partner (n=7/16, 43.8%) | | |
| Impacted decision to have children | Participants described deciding not to have children or not have any additional children as a result of MELAS, and concerns of passing MELAS on to children, possibly with a higher level of severity. One participant and caregiver also spoke about the difficulty of having children because of the symptom and impact burden of MELAS, such as increased sleeping and sound sensitivity. | 6 (37.5%) |
| Marriage hardships | Participant reported experiencing difficulties in their marriage as a result of MELAS but did not provide a description | 1 (6.3%) |
| Household chores/responsibilities (n=6/16, 37.5%) | | |
| Difficulty doing chores | Participants described having difficulty with chores such as cooking, running errands, taking care of pets, and cleaning. Five participants attributed difficulty doing to fatigue. | 6 (37.5%) |
| Cognitive function (n=3/16, 18.8%) | | |
| Difficulty remembering names | Participants described being unable to remember the names of both acquaintances and celebrities; one participant noted they will occasionally remember the name much later | 2 (12.5%) |
| Difficulty multi-tasking | Participant reported having difficulty doing two tasks at once, such as watching TV and listening to someone speak, but did not provide further description | 1 (6.3%) |
| Financial (n=3/16, 18.8%) | | |
| Affected income | Participant reported being on disability and the impact on their income, but did not provide a description | 1 (6.3%) |
| Cost of treatment | Participant described paying hundreds of dollars per month for the multiple medications needed to treat their MELAS medications, as well as their daughter’s MELAS medications | 1 (6.3%) |
| Difficulty qualifying for disability | Participant described difficulty qualifying for disability due to the rare and invisible nature of MELAS | 1 (6.3%) |
| Increased need for financial help | Participant described needing financial help from their spouse, who in turn also had a financial impact as a result of the participant’s MELAS | 1 (6.3%) |
| School impacts (n=2/16, 12.5%) | | |
| Difficulty participating in school activities | Participant described having difficulty in high school and college, with both extra-curricular activities and classes themselves, needing additional time or not being able to complete given tasks. The participant attributed difficulty participating in school to fatigue. | 1 (6.3%) |
| Reduced ability to finish school tasks | Participant did not provide a description of reduced ability to finish school tasks | 1 (6.3%) |
| Self-image (n=1/16, 6.3%) | | |
| Discrepancy between appearance and self-image | Participant described the mental burden of knowing their appearance does not reflect the reality of their disability | 1 (6.3%) |

^*^Concept reported by study participant

^†^Description of concept based on study participant report
